# Designing, Developing, Evaluating, and Implementing a Smartphone-Delivered, Rule-Based Conversational Agent (DISCOVER): Development of a Conceptual Framework

**DOI:** 10.2196/38740

**Published:** 2022-10-04

**Authors:** Dhakshenya Ardhithy Dhinagaran, Laura Martinengo, Moon-Ho Ringo Ho, Shafiq Joty, Tobias Kowatsch, Rifat Atun, Lorainne Tudor Car

**Affiliations:** 1 Lee Kong Chian School of Medicine Nanyang Technological University Singapore Singapore Singapore; 2 School of Social Sciences Nanyang Technological University Singapore Singapore Singapore; 3 School of Computer Sciences and Engineering Nanyang Technological University Singapore Singapore Singapore; 4 Institute for Implementation Science in Health Care University of Zurich Zurich Switzerland; 5 School of Medicine University of St Gallen St Gallen Switzerland; 6 Centre for Digital Health Interventions Department of Management, Technology, and Economics ETH Zurich Zurich Switzerland; 7 Future Health Technologies Programme Campus for Research Excellence and Technological Enterprise Singapore-ETH Centre Singapore Singapore; 8 Department of Global Health & Population, Department of Health Policy & Management Harvard TH Chan School of Public Health Harvard University Cambridge, MA United States; 9 Department of Global Health and Social Medicine Harvard Medical School Harvard University Cambridge, MA United States; 10 Health Systems Innovation Lab Harvard TH Chan School of Public Health Harvard University Cambridge, MA United States; 11 Department of Primary Care and Public Health School of Public Health Imperial College London London United Kingdom

**Keywords:** conceptual framework, conversational agent, chatbot, mobile health, mHealth, digital health, mobile phone

## Abstract

**Background:**

Conversational agents (CAs), also known as chatbots, are computer programs that simulate human conversations by using predetermined rule-based responses or artificial intelligence algorithms. They are increasingly used in health care, particularly via smartphones. There is, at present, no conceptual framework guiding the development of smartphone-based, rule-based CAs in health care. To fill this gap, we propose structured and tailored guidance for their design, development, evaluation, and implementation.

**Objective:**

The aim of this study was to develop a conceptual framework for the design, evaluation, and implementation of smartphone-delivered, rule-based, goal-oriented, and text-based CAs for health care.

**Methods:**

We followed the approach by Jabareen, which was based on the grounded theory method, to develop this conceptual framework. We performed 2 literature reviews focusing on health care CAs and conceptual frameworks for the development of mobile health interventions. We identified, named, categorized, integrated, and synthesized the information retrieved from the literature reviews to develop the conceptual framework. We then applied this framework by developing a CA and testing it in a feasibility study.

**Results:**

The Designing, Developing, Evaluating, and Implementing a Smartphone-Delivered, Rule-Based Conversational Agent (DISCOVER) conceptual framework includes 8 iterative steps grouped into 3 stages, as follows: design, comprising defining the goal, creating an identity, assembling the team, and selecting the delivery interface; development, including developing the content and building the conversation flow; and the evaluation and implementation of the CA. They were complemented by 2 cross-cutting considerations—user-centered design and privacy and security—that were relevant at all stages. This conceptual framework was successfully applied in the development of a CA to support lifestyle changes and prevent type 2 diabetes.

**Conclusions:**

Drawing on published evidence, the DISCOVER conceptual framework provides a step-by-step guide for developing rule-based, smartphone-delivered CAs. Further evaluation of this framework in diverse health care areas and settings and for a variety of users is needed to demonstrate its validity. Future research should aim to explore the use of CAs to deliver health care interventions, including behavior change and potential privacy and safety concerns.

## Introduction

### Background

Conversational agents (CAs) are computer programs that use text, speech, and other input modalities to enable communication with users [1]. They can be accessed through a variety of ways, such as social media platforms (eg, Facebook Messenger), websites, and smartphone apps, or deployed using stand-alone digital devices (eg, Alexa, Google Assistant, and Siri). The interactive nature of CAs makes them acceptable to a diverse group of users [2-4] and a preferred tool in a number of disciplines, including customer service, retail, and e-commerce [5-7].

In health care, CAs are increasingly used to assist in various tasks, such as patient education, self-management of chronic conditions, and routine task automation (eg, appointment booking), and support health professionals’ decision-making for diagnosis and triage [3,8-10]. More recently, CAs have seen large-scale implementation with the introduction of Babylon’s artificial intelligence (AI)–based symptom checker CA to the UK National Health Service and to Rwanda’s National Health Insurance Scheme [11]. CAs have the potential to support health care delivery, improve access to health care services, and automate tasks [12], and they may also reduce health professionals’ workload [13].

CAs vary in complexity and capability. There are 3 design dimensions used to classify CAs: purpose, communication channels, and response generation architecture [6]. According to *purpose*, CAs can be classified into *task- or goal-oriented CAs*, which respond to a limited number of tasks within a prespecified domain, or *non–task- or non–goal-oriented CAs*, which are potentially able to respond to an unrestricted variety of user requests [6]. *Communication channels* can commonly be divided into 2 main types: *text-based* or *voice-based* CAs. *Response generation architecture* can be broadly classified into 3 groups: *rule-based* and *retrieval-based CAs*, which produce a response by selecting it from a pool of predetermined responses either following simple rules to match phrases or identifying specific keywords in the text [6,14,15], and *generative-based CAs*, which use AI algorithms to develop a contextual response informed by the system’s previous and ongoing learning [6,14-16]. Although all 3 groups may involve the use of AI algorithms [6], rule-based CAs allow developers greater control over the conversation content and flow, which is a useful feature when developing CAs for health care. By contrast, AI algorithms, particularly neural networks, may develop decisions that are not explainable or understood by the end user, a phenomenon referred to as the *black box* [17]. In health care settings, the *black box* effect may lead to biased or erroneous decision-making and patient harm [18], which may limit the use of AI. A new field of explainable AI is currently emerging that aims to provide justification for algorithm predictions and increase system transparency, although the validity of results for individual patients should be carefully considered [19].

CAs can be deployed using a variety of digital devices, including smartphones. The widespread availability of smartphones in high-income countries and increasingly in low- and middle-income countries [20] makes them an ideal interface to deliver CA interventions. Smartphones offer users the possibility of continuous and dynamic monitoring of health conditions in a private space and at the time of their convenience [21] not only of subjective, self-reported data but also of objective, sensor-based data. Furthermore, smartphones allow for the delivery of interventions according to user needs [22]. CA interventions are complex and often require lengthy, costly design and development processes led by multidisciplinary teams of health care professionals, computer scientists, and app developers, which may limit the number of teams able to engage in CA development, particularly in low- and middle-income countries. However, mobile health (mHealth) interventions, particularly SMS text messages delivered using mobile phones, are effective in delivering health care interventions, especially in low-resource settings [23,24].

Several frameworks for the design and development of mHealth interventions currently exist, offering guidance at every step of the cycle, from the conceptualization of user needs [25,26] to the development of the digital health intervention [25-27]. These frameworks focus on generic, app-based interventions without a conversational interface. However, Zhang et al [28] described a framework for the development of AI-based CAs to deliver behavior change interventions that may require significant deployment of resources, including a large, multidisciplinary team, and close supervision of the AI algorithms to prevent unintended and potentially harmful effects on the users. However, to date, no conceptual framework for the design, development, and evaluation of rule-based CAs has been published despite a growing interest in the use of CAs in health care settings.

### Objectives

CAs constitute a specific type of digital intervention characterized by the use of a conversational interface, often led by an agent with a distinct personality as evidenced by its tone of speech, method of interaction, and visual representation, which is often associated with higher levels of engagement with the user. These features and the ubiquity of smartphones support the need for a framework that is accessible to large as well as smaller research teams with limited resources to guide CA development, including the distinct design and development challenges of CAs such as the creation of dialogs and the look and personality of the agent, grounded in current best evidence. Therefore, this research aimed to develop a conceptual framework for the design, development, evaluation, and implementation of smartphone-delivered, rule-based, goal-oriented, and text-based CAs for health care.

## Methods

**Figure 1:**
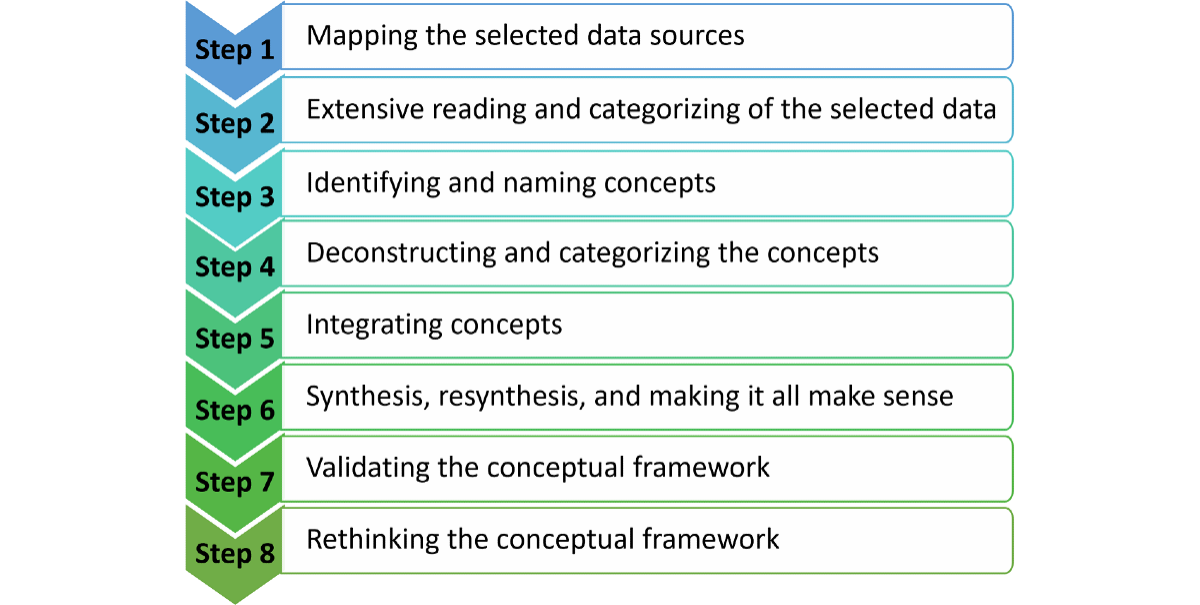
The 8 phases of the methodology by Jabareen [29] for conceptual framework development.

We developed the Designing, Developing, Evaluating, and Implementing a Smartphone-Delivered, Rule-Based Conversational Agent (DISCOVER) conceptual framework according to the methodology described by Jabareen [29], consisting of the iterative, qualitative analysis of multidisciplinary data based on the grounded theory method. It comprises 8 interlinked steps aimed at integrating and analyzing the data and developing and validating the conceptual framework [29] (Figure 1).

### Step 1

We conducted 2 literature reviews. The first review aimed to summarize the current literature on conceptual frameworks for the design, development, and evaluation of mHealth interventions, and the second review focused on smartphone-delivered, rule-based CAs. A description of these literature reviews can be found in Multimedia Appendix 1 [5,30-62] and Multimedia Appendix 2 [5,30-62]. Multimedia Appendix 3 presents the search strategy used to retrieve the studies for the review of CAs.

### Step 2 and Step 3

The screening of retrieved citations was performed in 2 stages, independently and in parallel, by DD and LM. The same 2 reviewers extracted data from all the included studies independently and in parallel. At all stages of screening and data extraction, the results were compared, and discrepancies were resolved by consensus between the reviewers.

### Step 4

The data analysis followed qualitative meta-synthesis to systematically summarize the findings across all the included studies. This step involved grouping the concepts extracted from both literature reviews into overarching domains.

### Step 5 and Step 6

The next 2 steps involved linking the overarching domains and developing the first iteration of the conceptual framework.

### Step 7 and Step 8

The conceptual framework was further amended based on discussions among the research team members and feedback from colleagues collected in a seminar. We subsequently applied the conceptual framework to develop a rule-based, text-based, smartphone-delivered CA prototype (*Precilla*) designed to support healthy lifestyle changes and educate participants about diabetes. The development, feasibility, and acceptability of Precilla have been reported elsewhere [63,64].

The feedback received from team members and colleagues and the lessons learned during the application study led to the refinement of concepts and domain labels, definitions, order, and grouping that were derived in the current version of DISCOVER presented in this paper.

### Ethical Considerations

This study was approved by the Nanyang Technological University Institutional Review Board (IRB-2018-11-032).

## Results

### A Framework for Guiding the Design, Development, Evaluation, and Implementation of Smartphone-Delivered, Rule-Based CAs in Health Care: Overview

The conceptual framework development was informed by the 2 literature reviews and iterative consultations within the research team. Further refinements were also informed by the development of our CA prototype (*Precilla*) [63,64] as well as by presentations at clinical seminars and conferences. Multimedia Appendix 4 outlines the methodology applied in the development of the DISCOVER framework according to each step described by Jabareen [29]. Multimedia Appendix 5 [63,64] presents the steps to develop CA Precilla mapped to the steps of the current version of the conceptual framework.

The 2 literature searches retrieved a total of 55 studies, of which 41 (75%) described conceptual frameworks for the design, development, and evaluation of mHealth interventions and 14 (25%) were clinical trials evaluating smartphone- and rule-based CAs. The findings from these reviews are presented in Multimedia Appendices 1 and 2. The “Characteristics of included studies” tables are presented in Multimedia Appendix 6 [47-58], Multimedia Appendix 7 [5,32,65-67], and Multimedia Appendix 8 [3,30,31,33,34,68-80].

**Figure 2:**
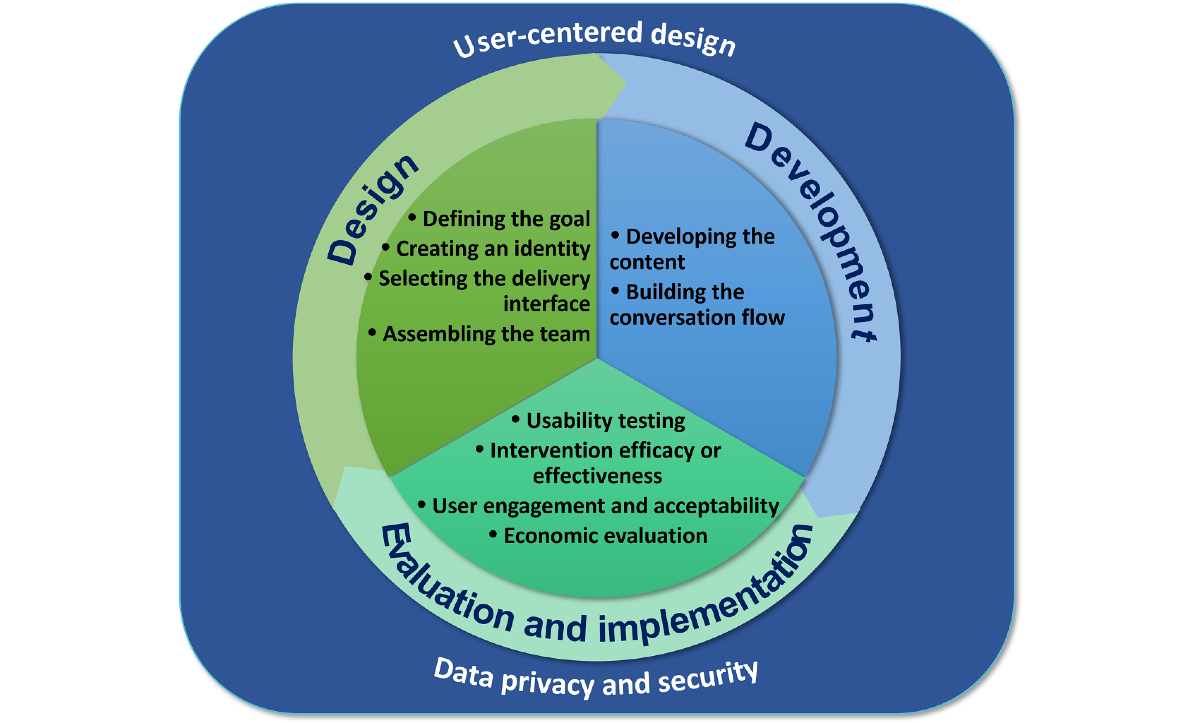
The DISCOVER conceptual framework for the design, development, and evaluation of rule-based, smartphone-based conversational agents in health care.

The initial framework contained 8 steps. They were subsequently condensed into 5 steps augmented by 2 overarching themes relevant to all phases of the development process. Further refinements led to the framework presented in this paper consisting of an iterative process of design, development, evaluation, and implementation steps, each comprising several components, as presented in Figure 2 and described in the following sections.

### Step 1: Design

The first stage comprised 4 interlinked steps encapsulating the initial conceptual work of identifying the health care focus of the CA, target users, multidisciplinary team members, and the CA delivery interface.

#### Defining the Goal

##### Overview

A clearly defined goal is the first step in the design process and the foundation that will guide the development and evaluation of the CA. This step consists of 3 interlinked areas of evaluation—completing a thorough needs assessment, defining the aim, and characterizing the end user and objectives—which, in turn, determine the parameters to be tested and reported. The CA goal was described in 64% (35/55) of the papers in our reviews [3,5,25-28,65,68-76,81-99].

##### Needs Assessment

The design process should commence with an in-depth needs assessment to understand existing gaps that may be filled by the CA. These may be informed by a literature review [83,90,91,96,100] to assess potential research areas and the needs and challenges of the target population, including not only patients but also caregivers, health care providers (HCPs), and other experts [25,26,87,89,95,98]. Researchers should also involve end users in this initial phase by using surveys and a variety of qualitative methods [70,87] such as in-depth interviews and focus group discussions to gather their views.

##### The Aim

Aligned with the needs assessment, the design team should formulate clear, attainable, and relevant objectives to drive the CA design and development process. It is important to consider the CA temporal profile, which characterizes 4 types of CAs according to the type and frequency of CA-user dialogs [101]. The CA temporal profile will also determine the type of objectives included, broadly classified as short term or long term [101]. A short-term objective refers to an outcome to be completed as soon as the interaction with the CA ends, such as medication reminders [30]. A long-term goal would involve several CA-user interactions being completed over a period, as in mental health interventions to promote mental well-being in the general population [3] or young people with cancer [70]. Complex CA interventions may include short- and long-term goals, such as CA Vik [30] providing medication reminders (short-term goal) and health education (long-term goal) to patients with breast cancer. Furthermore, Kowatsch et al [73] used prompts and reminder SMS text messages to enhance children’s discipline and routine, which are essential for the self-management of asthma.

##### Determining the End User

The next important design consideration is to determine the target population. An initial assessment should establish whether the CA will be offered to healthy users or individuals with a specific medical condition, caregivers, or HCPs. It is important to generate a detailed and accurate portrayal of the target user, including gender, age group, cultural beliefs and socioeconomic concerns, digital and health literacy, access to digital devices, and smartphone penetration rate. If the intervention is educational, a knowledge test should be implemented [73]. The acceptability of CAs by the target population and the perceived risk of using a CA for health care matters should be evaluated, particularly for severe or highly stigmatizing conditions [102] such as mental health disorders [103,104].

#### Creating the CA Identity

This step involves determining the CA’s name, appearance, tone of communication, language, and other characteristics that define its identity. This step was discussed in 25% (14/55) of the papers in our reviews [5,31,32,66,69,70,72-78].

##### CA Personality

User interaction with CAs appears to be enhanced when the CA displays a well-defined, positive, and empathic personality [105,106]. In general, giving a name and profile picture to the CA may enhance its social presence and user acceptance [107], although its effect appears to be small [106]. In health care settings, using a human-like avatar rendering realistic features, including medical attire, may increase user satisfaction [105], although avatars displaying highly realistic features may upset users and decrease engagement, an experience referred to as the “uncanny valley” [108].

Studies have consistently shown that CAs displaying empathy, relational behavior, and self-disclosure enhance the user experience [78,105] and increase the working alliance [109]. Conversely, users would notice if the CA did not convey empathy [69].

Acceptability may be further enhanced if the CA design acknowledges the specific cultural or demographic traits of the target population [73] or offers options to personalize the interface (eg, offering a male and female persona) [31,73]. Alternatively, CAs may explicitly disclose their identity [70] to reduce user expectations about their capabilities. Finally, CA personality should align with its intended function. For example, health care CAs often display one of two personality types: a more approachable, empathic coach-like personality, particularly if delivering behavior change interventions [77] supporting self-management of chronic disorders [73,78] and mental health conditions [3], or a health care professional persona to emphasize the legitimacy of the CA and its content [1].

##### Tone and Language

The language recommended for text-based interventions should be encouraging, positive, friendly, polite, and light-hearted and may include light humor while at the same time being formal [110]. To maintain the flow of the conversation, it may be advisable to use visual cues such as successive moving dots signaling that the CA is “typing” the next message.

The text should be written in clear, short sentences using simple language and avoid scientific jargon. The National Institutes of Health recommends that patient education materials be written at or below the sixth-grade reading level (ages of 11 and 12 years) to reach a diverse range of individuals with varying levels of literacy [111]. The readability of the text can be assessed using a scale such as the Flesch-Kincaid grade level to determine its suitability [112]. Furthermore, the CA should use the target population’s native language in its communications [75] and, if needed, the conversational content may be translated to one or more languages, particularly if the CA will be deployed in multiethnic, multilanguage societies.

With regard to the tone of the conversation, despite the text-based nature of the CA, it may be advisable to simulate more casual, verbal speech while avoiding the use of “textese” [113], a form of abbreviated written or typed language characterized by unconventional spelling and grammar (eg, “tonite” instead of “tonight”) and abbreviations and contractions (eg, “pls” instead of “please” or “wanna” for “want to”) [114]. Furthermore, words written in full capital letters should be avoided as they equate to shouting [110].

Emojis may be used to articulate emotions or other expressions more efficiently than text [70]. However, emojis are vulnerable to varied interpretations across cultures and contexts and should be used mindfully. Fadhil et al [115] noted a context-specific nature of emojis whereby they increased efficacy in a mental health intervention but did not help in promoting physical well-being.

CAs designed to address sensitive topics such as HIV and AIDS, sexually transmitted infections, or mental health disorders may emphasize the confidential nature of the messages or include code words to protect users’ privacy. This is particularly relevant in low- and middle-income settings, where family members may share a single smartphone [116].

#### Selecting the Delivery Interface

##### Human Involvement

Conditional to the CA’s aim, the design and development team may consider a “hybrid” intervention where the interaction with the CA would be complemented by regular interactions with HCPs offering timely feedback on a self-management technique or regular support and motivation [33,72,73]. Alternatively, as presented in the study by Stasinaki et al [79], the CA may be fitted with multiple channels, where the user can converse with the CA in one channel and directly with an HCP in another.

Peer support is recognized to play an important role in promoting adherence to self-management interventions [117] and a further point of human involvement to be considered. The CA intervention may include an additional communication channel for users to interact, share experiences, and receive peer support. For example, Wang et al [75] developed a WeChat intervention to support smoking cessation where the CA not only responded to individual users but also acted as a group moderator.

##### Delivery Channel

CAs may be delivered through a variety of channels, such as stand-alone apps [3,73] and existing messaging platforms [68,71,75] such as Facebook Messenger, Telegram, WeChat, and WhatsApp, or embedded in a website [69]. Each channel possesses its own set of complexities, and the decision regarding the delivery channel should be based on the target population needs and the expertise of the CA development team [118]. If the research team does not include app developers or computer scientists, the CA may be embedded in a messaging platform or may be developed using a CA development platform that offers templates or other design solutions for individuals with no previous programming knowledge [5,118], such as Chatfuel, ManyChat, and others. CAs are generally web-based, and some of these platforms are free of charge. Alternatively, if the team expertise or project budget allows, the CA may be delivered through a stand-alone app. This approach offers design flexibility, such as a variety of data collection sources including smartphone sensors, health programming interfaces, connected medical devices, and patient self-reported data [119]. The combination of subjective patient reports with objective, real-time data may reduce users’ responsibility to update their progress and at the same time receive relevant, dynamic coaching based on the current data [120], which in turn may increase adherence to the intervention.

In addition, factors associated with the target population may also affect the selection of the most suitable delivery channel and operating system (eg, Android or Apple’s iOS). For example, Kamita et al [71] implemented their CA on the messaging platform “LINE” as it was the most popular social network service in Japan, and Wang et al [75] selected WeChat, the most common messaging app in Hong Kong.

##### Communication Modalities

Aligned with the framework focus, text would be the CA’s main input and output modality. Messages should be brief, fit the mobile screen without scrolling [69], and be of an adequate font size to allow for comfortable reading. Moreover, if the CA targets populations for whom reading might be challenging, such as older adults or visually impaired individuals, text-to-speech assistive technology may be incorporated into the app.

Visual aids such as images or videos are useful to adapt content to audiences with lower educational attainment [121], deliver personal narratives relevant to the end users (eg, young people with cancer), or decrease the amount of textual information [76]. When using multimedia content, it is important to use high-resolution files to avoid pixelated or blurred images. Furthermore, if pictures are obtained from the web, developers should abide by copyright regulations and either source the pictures from free stock photo repositories, acquire the image rights, or produce the images in-house.

#### Assembling a Multidisciplinary Team

The composition of the design and development team would be based on the objectives of the intervention. In addition to the inclusion of health professionals with the relevant expertise, it is recommended to include end users as well [69,70]. For example, a CA to support a lifestyle intervention in overweight adolescents was developed by a multidisciplinary team including computer scientists, physicians, a psychotherapist, and diet and sports experts [72]. End-user involvement in the intervention design is critical to ensure that it aligns with user needs. User involvement was reported in a large number of studies in our review (36/55, 65%); for example, young people with cancer participated in focus groups to refine the content of a CA aimed at delivering positive psychology to enhance well-being [70], and young patients with asthma and their parents were part of a multidisciplinary team of experts who developed a CA to improve cognitive and behavioral skills [73]. In general, studies that mentioned the composition of their multidisciplinary teams often reported computer scientists and physicians as key members [72-75], although other health professionals such as physiotherapists [78], psychologists [3], and music therapists [76] may be included as well.

### Step 2: Development

#### Developing the Content

Content development may involve determining the sources of information, adapting content to the target audience, defining the behavior change theories and techniques guiding the intervention [28,94], and establishing error management and safety-netting strategies [26-28, 30, 67-70, 73, 75, 77, 79, 80, 82, 83, 85, 87, 90, 92-97, 99, 100].

##### Evidence-Based Information

All health-related information included in the CA should be derived from reputable sources and adequately referenced. Sources of evidence-based information include comprehensive literature reviews; clinical practice guidelines; Cochrane systematic reviews; and reputable organization websites such as the World Health Organization, MEDLINE Plus, and the Centers for Disease Control and Prevention in the United States or the National Health Service Health A to Z in the United Kingdom [65]. For example, Kowatsch et al [73] used evidence from multiple sources such as published literature on the improvement of asthma management in children [122], technology acceptance research [123], and user-CA working alliances [124] to inform their intervention for asthma management.

##### Managing Errors

Another important aspect of content development is to ensure an adequate understanding of user requests, particularly for potentially serious or life-threatening health conditions. Safeguards to be implemented within the dialog include the request for clarification if the CA receives an unfamiliar input or directing the user to contact an HCP or a human administrator [125,126]. These strategies were included in TensioBot, an intervention to facilitate self-measurement of blood pressure where, after obtaining confirmation of a blood pressure measurement value outside the normal range, the CA alerted the attending physician [68]. Important strategies to manage unintended errors include using validated data entry fields; limiting the data input to predetermined number ranges, words, or characters; or including predefined options for the user to select.

##### Safety Netting

In general, health care CAs should include a disclaimer clearly stating that the intervention “does not replace healthcare provider’s advice.” Furthermore, in the case of health conditions associated with rapid deterioration of patient status leading to medical emergencies, such as cardiovascular conditions, diabetes, chronic pulmonary disorders, or mental health conditions that increase the risk of suicide, information should be included to assist users in managing an emergency situation, such as the provision of emergency services or crisis helpline telephone numbers [127], links to contact their primary physician, or clear advice on first aid treatments such as offering a sugary drink to manage a hypoglycemic event in a person with diabetes [128].

##### Types of Messages

The content and style of the messages should be aligned with the health condition and CA aim. Broadly, the messages may be educational [30,78] or motivational [34,77,79] or deliver reminders to perform a self-management task [68], input data [77], comply with preset tasks [73], take a medication, or attend an HCP appointment [68]. For CAs tasked with engaging with the user during clinic visits, it may be useful to include a status report or summary of the consultation [126].

CAs assuming a coach-like persona might emphasize sympathy, empathy, and participants’ achievements [78]. Interventions attempting to modify users’ behavior may deliver messages with higher emotional content, as reported in the study by Carfora et al [80], where only emotional messages led users to reduce red meat consumption. In addition, the Wang et al [75] CA used 4 types of messages to deliver a smoking cessation intervention: group announcements, health-related information, reminders to share positive results and progress, and fixed answers to frequently asked questions or requests.

##### Behavior Change Theories

CAs are increasingly used to promote behavior change [1,129]. Behavior change interventions are complex [130] and often comprise one or more behavior change techniques (BCTs) to induce change. In our assessment, 4% (2/55) of the studies used a behavior change theory to guide the intervention design, including the Health Action Process Approach [78] and the technology acceptance model [71]. In addition, 13% (7/55) of the studies [31,72-75,77,80] reported the use of specific BCTs such as goal setting, self-monitoring, tracking and feedback, social support, use of rewards, and anticipated regret.

For example, a study described a multicomponent behavior change intervention incorporating several BCTs, such as goal setting, self-monitoring, stimulus control, and behavioral contract, to support a healthy lifestyle for adolescents with obesity [34,79]. Furthermore, including group chats where peers or HCPs offer relevant information and emotional support may also assist in promoting positive behavior change, such as using a CA-led WeChat peer group to promote smoking cessation [75].

##### Optional Add-ons

Depending on the purpose of the CA, it may be appropriate to integrate data from external devices such as glucometers [131] or activity trackers [119]. Alternatively, access to smartphone sensor data [132] may facilitate passive monitoring of the user’s activity [79] or determine novel digital biomarkers to assess the user’s mood [133] or disease status [134]. The use of smartphone sensors for passive monitoring may further allow for real-time information sharing with HCPs, caregivers, or peers, a feature that may be particularly useful to monitor older people living alone, who may be at higher risk of falling, or individuals with severe chronic illnesses and multiple hospital admissions.

#### Building the Conversation Flow

A good CA is eloquent and knowledgeable and, thus, requires a meticulously crafted script. Conversation flow building was discussed in 35% (19/55) of the papers in our literature search [3,27,28,30-32,65,73,78,79,82,85,87,92-96,99].

##### Providing Suitable Answer Options

For a good conversation flow, the predefined answer options should be sufficient and appropriate to align with the user intent, defined as the user goals or intentions in each conversation turn. Constructing a mind map outlining the different facets associated with a topic (eg, medication adherence) and the likely influencing factors (lifestyle components or emotional state) would help predict the most relevant answer options to provide to the user [135].

##### Selecting a Mapping Tool

A mind map is a diagram representing concepts, ideas, or tasks generated from a key concept, which is generally represented in the center of the graph [136]. Mind maps are an effective method of brainstorming [137] that can be applied to building the conversation flows. Several web-based programs and platforms are available to organize the conversation flow, including tools specifically designed to build the CA conversation, such as SAP Conversational AI [138] or MobileCoach [35]. Conversation flows may also be built using nonspecific mind mapping software such as Xmind [139]. Mind mapping is useful to assist in recording the flow of conversations between different topics or different user interactions. A well-constructed conversation flow leads the conversation, guides the user, and can address all relevant questions about its purpose. Furthermore, interactivity, personalization, and consistent messaging have been noted as valued qualities [140].

##### Personalizing Content and Delivery

Interventions should be tailored to individual participant needs [110]. When compared with generic CAs, context, situational, or individually aware agents promote a more positive user experience [132]. Personalized interventions include addressing the user by their name or nickname [141]; delivering notifications and reminders tailored to individual needs [110], such as medication or appointment reminders; and notifications for missed activities or unread messages [30,78]. For example, an intervention promoting self-management of chronic pain offered personalized content based on the user’s type and duration of pain and personal interests [78].

An important caveat involves the design of interventions offering personalized advice based on user measurements, such as suggesting a treatment based on individually reported data (eg, blood glucose levels or blood pressure readings), as these interventions may require regulatory oversight and be considered a “mobile medical application” [142].

##### Selecting Appropriate Message Timing and Frequency

The timing and frequency of messages are important components when planning the intervention and may be determined by the intervention scope as well as user preference. Earlier studies on SMS text messaging interventions have suggested a preference for weekly messaging [113]. However, different intervention types may require a more adaptive message delivery system, such as smoking cessation programs that often require an increased volume of messages close to the desired quit date [143] or high-risk behavior prevention programs targeting binge drinking or inappropriate sexual behaviors timing their messages to when the risky behavior is expected to occur, for example, on a Friday night [116,141]. Therefore, strategies for message delivery and frequency could be adapted to suit the CA intervention.

Just-in-time adaptive interventions (JITAIs) leverage smartphone sensor data to “provide the right type (or amount) of support at the right time” [22]. Smartphone sensor data would determine and even predict “states of vulnerability” (susceptibility to negative health outcomes) [144] and “states of receptivity” (the capacity to receive, process, and use the intervention) [120] in the user when the intervention may be required and more useful. This novel approach may be particularly useful for behavior change interventions supporting a healthy lifestyle, such as increasing physical activity or adhering to a healthier diet, or supporting substance use remission [22,120]. Nevertheless, researchers considering this approach should take into account human and economic resources as JITAI design may require a larger development team that includes computer scientists and app developers.

##### Using Engagement Strategies

Strategies to keep the users engaged for the intended duration of the intervention are particularly important in health care settings. These aspects were discussed in 11% (6/55) of the studies in our reviews [3,30,31,73,78,79]. Reported strategies included notifications, weekly summaries, reminders, motivational statements, persuasive techniques, a high frequency of messages to promote habit formation, and daily encouragement. In addition, CA-specific engagement strategies included building rapport and attachment with the user [72,73] or adding gamified components to incentivize CA use for rewards and points [73,79].

### Step 3: Evaluation and Implementation

#### Evaluation

The evaluation of digital interventions, including CAs, starts early in the development process and comprises several iterative steps. To ensure the validity of the results, the process must use a robust methodology that is adequate for the intervention design [15]. In digital health interventions, a commonly used evaluation methodology is the multiphase optimization strategy by Collins et al [145,146].

The CA evaluation follows 3 distinct stages representing the intervention development process. The initial iterations of the CA may be evaluated using one or more usability testing methods [147] aiming to produce a minimum viable prototype. Once this working prototype is ready, pilot and randomized trials may ensue to assess the effectiveness of the CA [148]. Several aspects of CA evaluation were discussed in 36% (20/55) of the studies in our reviews [25, 26,28, 33, 71,73, 83, 85, 86, 88, 89, 91-95, 97-100].

The evaluation design may include one or more aspects of the CA functionalities, including clinical or technical attributes and user experience. The outcomes should be clearly defined and include widely used and validated outcome measurement tools whenever possible to improve the comparability and reproducibility of the research results. Examples of outcome measurement tools include the Patient Health Questionnaire-9 [149] to screen for depression, the Flourishing Scale [150] to assess psychological well-being, the Brief Pain Inventory [151] to assess pain intensity and its interference in activities of daily living, and the Working Alliance Inventory-Short Revised [152] to evaluate the CA-user working alliance.

#### Usability Testing

The evaluation of the CA should start early in the development cycle [153]. In the initial stages, formative evaluation aims to assess the viability of the digital tool by assessing its usability, usefulness, and user experience [154] using one or more qualitative or quantitative research designs. Qualitative methods include surveys, interviews, focus group discussions, and “think aloud” protocols [147] in which users express their opinions about the product as they use it. Quantitative methods include closed-ended questionnaires, task completion assessments, and A/B testing [147,155]. An A/B test, split test, or controlled experiment compares two or more versions of a product to evaluate the intervention components that perform better or are preferred by the user [155]. This stage relates to the screening and confirming stages in the multiphase optimization strategy [145,146], which use a fractional factorial design to assess which components should be included in the digital intervention and the best dosages to use in a more cost-effective fashion. Finally, microrandomized trials are another novel methodology that is particularly useful for assessing and optimizing the delivery of JITAIs [156]. Microrandomized trials allow the randomization of multiple components to occur at multiple times triggered by predefined decision points [156] and have been used to evaluate CA interventions, as reported by Kramer et al [119,157].

#### Efficacy and Effectiveness of the CA Intervention

Once initial evaluations have determined the components that should be included in the intervention and the frequency of administration, a traditional randomized trial design should be implemented to assess the effectiveness of the CA intervention compared with current best practices [145,146,148]. Given the complexities and cost that a full-powered randomized controlled trial often entails, researchers may consider conducting a pilot study to refine the study methodology or assess the feasibility of the study design and participant recruitment strategies, among other aspects [158]. For example, Casas et al [77] conducted a pilot study to preliminarily assess a CA aimed at coaching participants to make healthier food choices, whereas Greer et al [70] evaluated a CA delivering a positive psychological intervention to young people with cancer.

#### User Engagement and Acceptability

##### Overview

Digital health interventions often report high rates of participant attrition, which may limit the validity of research findings and, more importantly, the effectiveness of the intervention. Therefore, the assessment of the CA-led intervention should be complemented by regular evaluations of end-user adherence to as well as engagement with and acceptability of the intervention. Several assessment methods are commonly used, including quantitative, data-driven analyses and qualitative assessments of users’ opinions.

##### Data-Driven Analyses

The definition of adherence to digital health interventions refers to the extent to which a user has interacted with the intervention [159]. This term may be used to define the degree to which a user interacts with the CA (greater adherence equals more time engaging with the intervention) or the degree to which the user-CA interaction complies with the prescribed recommendation (intended use of the intervention) [159]. In health care interventions, the concept of “intended use” is preferred, and it should be clearly defined during the CA design and development stage for the subsequent adherence measurements to be meaningful. Increased adherence to an intervention may be related to its increased effectiveness [75,160], although the data are not conclusive [3,161,162].

User engagement with the CA may be evaluated using data metrics such as the times the user opened the app, time spent interacting with the CA, the extent of the dialog, or the number of screens opened if the CA also includes other functions [70]. Chaix et al [30] measured use duration, interest in various educational contents, and level of interactivity as indicators of engagement. Nevertheless, researchers should consider the challenges of defining engagement with digital interventions, which may include other user-related variables such as the severity or stage of the disease as well as the long-term engagement with the CA [163].

Other aspects of CA use, such as underused or missing topics or CA functionalities not working as intended, may also be assessed. CA use analytics are often embedded in host platforms. Commercial platforms such as ManyChat [164] may offer a variety of built-in analytics tools such as the number of times the CA is accessed. Some of these platforms offer free-of-charge services. For health care CAs, the open-source MobileCoach platform [35] offers flexible, customizable use analytics.

##### Qualitative Evaluation

Acceptability refers to the “affective attitudes towards a new digital health intervention” [165]. It is a dynamic concept comprising the intention to engage with the novel CA, the actual interaction with the CA, and the postengagement satisfaction [165].

Acceptability is a subjective term that is generally assessed using questionnaires or other qualitative methods such as focus groups or interviews. For example, Kowatsch et al [73] evaluated the acceptance of a CA to support asthma self-management using a 7-point Likert scale (strongly agree-strongly disagree) for perceived usefulness, ease of use, enjoyment, and use intention, and Echeazarra et al [68] used a survey with questions on ease of use, preference for the CA over existing methods, CA usefulness for its intended purpose, and whether the user had stopped using it as measures of acceptability and satisfaction. Furthermore, Gabrielli et al [69] facilitated a participatory design workshop where suggestions for improvement were provided via open-ended questions, and Ly et al [3] conducted semistructured interviews on the benefits, opportunities, and challenges associated with the CA for mental health. Yan et al [166] described a very involved process of evaluation of an mHealth intervention to promote physical activity. A focus group discussion was organized whereby each SMS text message was displayed and participants were required to respond either with “Yes, I like it” or “No, let’s change it to make it better.” This voting was then followed by a discussion in which suboptimal messages were improved and the strengths of effective messages were noted. Finally, participants may also be questioned about their willingness to recommend the conversation to others, which is a good indicator of satisfaction and acceptability [70].

Several aspects of user engagement and acceptability may be measured using one of several app quality rating tools, of which the most commonly used one is the Mobile App Rating Scale [167]. The use of standardized, validated rating scales may improve the reproducibility of this research area and facilitate the reporting of trial results, although they are not specific for CAs.

#### Economic Evaluation

The economic evaluation includes not only the affordability of the project but also the cost-benefits associated with developing the CA. These analyses should consider the end-user perspective as well as the potential benefits for the health care system in general [168,169]. Digital health interventions appear to be cost-effective [170], although reports often present varying, inconclusive results [171]. Although it is often mentioned that one of the potential advantages of digital health interventions, particularly in the long term, may be a significant decrease in health care costs [172], the upfront expenses of developing the digital intervention might be substantial. For example, Kowatsch et al [73] reported upfront expenses of approximately US $250,000 to develop a CA to support asthma self-management in young patients. The development costs will vary conditional to the type and functionalities of the CA, the use of a messaging platform or development as a stand-alone app, and the number of team members, among other aspects. Despite the increasing importance of conducting economic evaluations of digital health care interventions, only 2% (1/55) of the studies included in our reviews reported economic evaluation data [73]. Recent documents from the World Health Organization [168] and the International Training and Education Center for Health [169] at the University of Washington in the United States, as well as a recent review [171], present a practical overview of how to perform economic evaluations.

#### Implementation

Once the effectiveness of the CA intervention has been determined in rigorous clinical trials, the research team should consider implementing the intervention in the broader population. Implementation research aims to integrate research and practice [173] and understand the users and context in which an intervention would be implemented. The research methods, including pragmatic trials, participatory action research, and mixed methods studies, aim to assess the intervention “acceptability, adoption, appropriateness, feasibility, fidelity, implementation cost, coverage, and sustainability” [174-177]. Important considerations include the need to upgrade the systems to adapt to higher traffic, personnel to provide long-term system maintenance and updates, and the costs these changes may incur [25,26]. Furthermore, the team should consider CA intervention commercialization strategies, including engaging HCPs, health insurers, or governmental organizations if aligned with the health care focus of the intervention [26].

Finally, the team should be aware of and comply with the current regulatory frameworks for digital health interventions. Increasingly, countries are developing national policy frameworks to regulate the evaluation, use, and commercialization of digital health interventions [178], particularly if the intervention is considered a digital therapeutic [179]. Digital therapeutics refer to “evidence-based therapeutic interventions that are driven by high-quality software programs to prevent, manage, or treat a medical disorder or disease” [179], may require a provider’s prescription to be accessed [179], and often require approval from official regulatory bodies such as the Food and Drug Administration in the United States [180] and the Conformité Européenne mark in the European Union (EU) [181].

### Cross-Cutting Considerations

The themes described in this section are relevant throughout all the design stages referred to in the previous sections.

#### User-Centered Design and Co-design

User-centered design refers to design practices that include the end users’ views to guide the process, either in a passive, consultive manner or as active participants in the design process (co-design) [182]. Several approaches to user-centered design have been described. They share the general principles of involving users during the design process, although the steps involved in the process and the type and extent of end-user involvement may differ. They include but may not be limited to human-centered design [183,184] and design thinking [185] (often considered synonyms), user-centered design [186], co-design [182], and participatory action research [187].

End users include patients, caregivers, HCPs, or other relevant stakeholders. There are several benefits of including end users as part of the CA development team, such as a better understanding of users’ and communities’ needs, development of culturally sensitive products, and improved communication between the different stakeholders [188,189]. This, in turn, may increase compliance with the intervention and improve health-related outcomes [190]. For example, to develop a CA to promote positivity and well-being in young people after cancer treatment, Greer et al [70] conducted interviews and focus groups with young adults treated for cancer to refine the informational content.

During the evaluation stage, thinking-out-loud usability testing is another example of a user-centered design methodology in the design of digital health interventions, including CAs [191].

The role of user-centered design in the development of digital health interventions has been repeatedly emphasized by several frameworks included in our review (36/55, 65%) [25,26,36-39,41-46,62,69,70,73,76,82-85,87-100,110].

#### Privacy and Security

##### Overview

Safeguarding the privacy and security of CA users’ data is essential and should be a part of the entire design and development cycle. Health information is considered personal, sensitive information that should be protected at all times. The level of data protection should align with the data collected by the CA, if any. Therefore, the functionalities of the CA will determine the type of sensitive data to be collected and guide the inclusion of data protection software such as firewalls and encryption.

In general, developers should minimize the amount of personal and sensitive information collected from users by asking specific questions to avoid oversharing or simply providing predetermined responses instead of using free text. Furthermore, all CAs should include a privacy policy that is brief and written in clear language outlining the data collected and the uses of these data. All data must be encrypted during transit (when the message is being sent) and at rest (when the message has been delivered) [192]. The platform on which the CA will be deployed may also vary according to the CA functionalities. For example, a CA collecting users’ personal data should not be deployed on proprietary or messaging platforms as the platform data management policies may not be clearly reported [65] or data sharing with third parties may occur without informing the user [193]. This might create an ever-increasing digital footprint, potentially allowing for user identification from data aggregation rather than actually identifiable information [194].

A 2020 framework for governing the responsible use of CAs in health care highlighted the importance of safeguarding data privacy, including user health data, history of interactions, and disclosure of user data even if unintended [195]. In addition, the framework highlighted the user’s right to access their personally identifiable information, the requirement of user consent before recording or saving health-related data, and the preclusion of using the stored data as a means of surveillance or to discriminate users against health care privileges or coverage [195].

##### Compliance With Data Privacy Laws

Health care CAs that collect users’ sensitive data must comply with country-relevant data privacy laws, such as the Health Insurance Portability and Accountability Act in the United States [192] or the General Data Protection Regulation (GDPR) in the EU [196]. These laws’ jurisdiction is generally limited to the issuing country; however, the GDPR applies to any EU citizen within or outside the EU. The GDPR, which went into effect in 2018, is an overarching law that aims to enhance the rights of individuals over their personal data, defined as any data that may allow for the identification of a person on their own or combined with other data, including pseudonymized data [196]. Alternatively, the Health Insurance Portability and Accountability Act is industry-specific and applies only to health-related data [197]. Other countries have adopted their own data protection laws and regulations. In Singapore, the Personal Data Protection Act is a baseline regulatory framework informing the collection, distribution, and use of personal data [198].

In addition to the aforementioned GDPR, children’s data are generally more stringently safeguarded. For example, in the United States, the Children’s Online Privacy Protection Act [199] requires that verifiable parental consent be obtained by all digital operators (not restricted to health care) collecting data from children (aged <13 years). Similar considerations are included within the GDPR and the Singapore Personal Data Protection Act, with the caveat that, in some European countries, parental consent is required for children and adolescents aged <16 years.

## Discussion

### Principal Findings

We present a new conceptual framework for the design, development, evaluation, and implementation of smartphone-delivered, rule-based, and text-based CAs. The DISCOVER conceptual framework includes 8 iterative steps arranged in three main groups: (1) design, which includes defining the goal, creating an identity, assembling the team, and selecting the delivery interface; (2) development, which comprises developing the content and building the conversation flow; and (3) evaluation and implementation. User-centered design and privacy and security were included as cross-cutting considerations, which are relevant at every stage of the framework.

This framework was based on the comprehensive analysis of 36 mHealth frameworks, 5 CA taxonomies, and 14 primary studies reporting on the design and development of rule-based health care CAs. The framework was applied in a web-based pilot study using a CA deployed on Facebook Messenger. The existing mHealth frameworks provided general guidelines to develop mHealth interventions for health care, from the characterization of the target population to evaluation, with emphasis on the application of user-centered design techniques in all stages of development. Concurrently, the CA taxonomies provided focused on several aspects of CA design and evaluation as well as the impact of design features on CA-user interactions.

Considering the multifaceted nature of embodied CAs, we decided to focus on CAs that are nonembodied.

### Comparisons With Prior Work

The existing frameworks for the design and development of mHealth interventions provide detailed guidance in all steps of the intervention development, starting with an understanding of the needs and the profile of the end users through a review of existing literature or formative research [67], and they emphasize the need for patient and public involvement to make the intervention as relevant to the target population as possible [90,98]. These frameworks also described the importance of conducting iterative evaluations to identify limitations before testing the mHealth intervention in a larger-scale trial [28,98,99]. However, the literature on the design and development of CAs was restricted to the development of taxonomies that were not limited to health care describing CA design platforms [5], classification of CAs according to the approach to conversation design [67], characteristics of embodied agents [66], or the impact of CA characteristics on user interactions [32]. Moreover, the taxonomy by Denecke et al [65] referred to health care CAs, but they focused exclusively on CA evaluation. Therefore, a conceptual framework guiding the development of health care CAs was needed to expand previous mHealth frameworks with elements particularly relevant to CAs, such as personality development, converting evidence-based content into conversations, and using novel research designs for evaluation. Furthermore, our framework focused particularly on the development of the CA, including personality, display of empathy, and disclosure of its identity as a computer-generated entity without human involvement, and on the development of dialogs guided by up-to-date evidence-based information sources.

This framework described the development of rule-based CAs, allowing the research team total control of the conversation and dialog flow. There are several reasons for this. First, our framework presents easy-to-follow steps that could be applied by smaller research teams that do not include computer science or AI specialists or that undertake the CA development project under restricted financial resources. Second, we aimed to provide guidance for the development of goal-oriented CAs aimed at delivering health education content or simple interventions aimed at improving healthy lifestyle choices or self-management behavior and, therefore, prioritize control over the conversation content using a rule-based paradigm, albeit less engaging, over AI algorithms that have yet to become truly explainable.

### Implications for Future Research

Future research should apply the DISCOVER conceptual framework to the development of CAs offering behavior change interventions aimed at different specialties, settings (hospital or outpatient), target groups, and cultures. Moreover, although the use of theories in the design of behavior change interventions is favored and may increase the effectiveness of the intervention [77,200], it is still unclear which behavior change theories or techniques are better suited for CA-led interventions. Alternatively, because of the interactive nature of CAs, it would be appropriate to assess whether behavior change interventions previously proved effective in traditional face-to-face settings are equally effective when led by a CA.

Although the concepts of identity creation, conversational flow, and delivery are important, their relative relevance to varying target populations is still unknown. In addition, more research on the assessment of health care chatbot interventions can help inform the ideal health-related outcome measures and digital data sets required for a comprehensive evaluation. Finally, although this framework is comprehensive and many components may apply to AI CAs, a separate framework is needed to describe specific aspects relevant to AI CAs, such as dialog development using machine learning or natural language processing techniques, voice versus text parsing, and many others.

### Strengths

This is, to the best of our knowledge, the first conceptual framework outlining the steps required to develop a smartphone-delivered, rule-based health care CA offering clear yet comprehensive guidelines to accommodate health care researchers with varying computer science expertise.

The DISCOVER framework builds on an analysis of existing mHealth frameworks and a stringent analysis of rule-based CA literature complemented by the team’s demonstration of its applicability in the development of a rule-based CA to support lifestyle changes in people at risk of developing diabetes.

### Limitations

Much of the information provided is anecdotal or derived from research conducted on SMS text messaging and other mHealth interventions because of the scarcity of research on the evidence-based development of rule-based CAs for health care. Therefore, this framework provides an overview of the main steps required to develop a rule-based CA.

The descriptions and examples presented in the conceptual framework focused on CA interventions for end users to support either a healthy lifestyle or the management of a chronic condition, as derived from the literature reviews and our experience developing a CA. Nevertheless, the design and development principles discussed in this study could apply to other relevant user groups such as caregivers and health care professionals.

Furthermore, this framework is focused on rule-based CAs and, although it may guide researchers in the development of particular aspects of AI CAs, it does not provide guidance on the development of AI-based conversations. In addition, the economic, social, and behavioral characteristics of different populations may limit its generalizability.

### Conclusions

The interest in and potential for CAs in health care are growing, but guidelines to design, develop, evaluate, and implement these interventions are currently lacking. Drawing on published evidence, the DISCOVER conceptual framework provides the first attempt to fill this void. The process was divided into 8 iterative steps arranged in 3 overarching groups and complemented by 2 cross-cutting considerations. Future research should explore aspects of CA development such as the use of behavior change theories and privacy and safety concerns. Further evaluation of this framework in diverse health care areas and settings and for a variety of users is needed to demonstrate its validity.

## Appendix

Multimedia Appendix 1 Literature review of conceptual frameworks for the design, development, and evaluation of mobile health interventions.

Multimedia Appendix 2 Literature review of smartphone-delivered, rule-based conversational agents.

Multimedia Appendix 3 Search strategy for the conversational agent research trial review.

Multimedia Appendix 4 Methodology implemented for conceptual framework development using the conceptual framework development steps described by Jabareen [29].

Multimedia Appendix 5 Mapping of the steps of the conceptual framework applied to the design, development, and evaluation of Precilla.

Multimedia Appendix 6 Design, development, and evaluation frameworks for mobile health interventions.

Multimedia Appendix 7 Classification systems for conversational agents.

Multimedia Appendix 8 Characteristics of clinical trials on rule-based conversational agents.

## Abbreviations

AI: artificial intelligence
BCT: behavior change technique
CA: conversational agent
DISCOVER: Designing, Developing, Evaluating, and Implementing a Smartphone-Delivered, Rule-Based Conversational Agent
EU: European Union
GDPR: General Data Protection Regulation
HCP: health care provider
JITAI: just-in-time adaptive intervention
mHealth: mobile health
