# Supplementary material for: Designing, Developing, Evaluating, and Implementing a Smartphone-Delivered, Rule-Based Conversational Agent (DISCOVER): Development of a Conceptual Framework
Source: JMIR Mhealth Uhealth. 2022 Oct 4;10(10):e38740. doi: 10.2196/38740 (PMC9579935; doi:10.2196/38740)
Supplement: Multimedia Appendix 1 [file mhealth_v10i10e38740_app1.docx]

**Multimedia Appendix 1**

Literature review of conceptual frameworks for the design, development, or evaluation of mHealth interventions

A series of literature searches in Medline (Ovid) were conducted in April 2019 and updated in December 2020 using the search terms “mHealth”, “texting”, “text messaging”, “short message service OR SMS”, “chatbot”, “conversational agent” AND “framework”, complemented by backward and forward snowball searches of included studies. mHealth defines the use of mobile phones, tablets, or other wireless devices to support healthcare delivery and public health [61]. The keyword “mobile health” was used in June 2022 to search for further studies published until December 2020. Studies were included if they were in English, published in a peer-review journal, and outlined a set of principles for design, development, or evaluation of mHealth interventions, including smartphone apps, text messaging, or CA-based interventions. Eligible studies should discuss the development of a new framework or the use of one or more existing frameworks. Data extraction included the name, aims, steps, and application of the included mHealth frameworks. The search, screening, and data extraction were performed in parallel by two reviewers (DD and LM). Any discrepancies were resolved through discussion and consensus. Data were extracted and analyzed using qualitative thematic analysis [60].

**Results**

The initial search retrieved a total of 749 citations, of which 21 studies were included. The updated searches in December 2020 resulted in an additional 7 studies, while a June 2022 update to include the keyword “mobile health” added 13 extra papers, for a total of 41 included studies. (Figure S1).

**Figure S1.** mHealth framework studies selection flowchart

A total of 36 studies reported on conceptual frameworks for the design, development, or evaluation of mHealth interventions, while five studies described several taxonomies to classify, design, or evaluate CAs. Multimedia Appendix 6 presents a summary of studies describing the mHealth intervention design, development, or evaluation frameworks and Multimedia Appendix 7 summarizes the CA taxonomies.

#### mHealth frameworks

The identified frameworks guided the design, development, or evaluation of mHealth interventions: smartphone apps (n=25, 69%), text messaging (n=6, 17%), interventions delivered in more than one platform (n=4, 11%). and CAs (n=1, 3%)

A total of 22 studies (61%) [37,38,39,40,41,42,43,44,46,62,82,84,85,88,89,90,92,94,95,98,100,110] applied one or more pre-existing frameworks, while 11 studies (31%) [25,26,27,28,45,81,87,91,96,97,99] reported the development of a new framework. In three studies (8%) [36,83,93] the origin of the framework was unclear. The mHealth frameworks targeted a variety of healthcare topics including behavior change interventions (n=15, 42%) supporting healthy lifestyle change [26,27,28,37,38,43,44,46,82,93,99,100,110], smoking cessation [94], reduction of alcohol consumption [41] or behavior change in general [43]. Other healthcare topics were: chronic disease self-management (n=7, 19%) [83,87,88,89,92,96,98], mental health conditions (n=5, 14%) [25,81,84,91,95], healthcare delivery improvement for hard-to-reach areas (n=3, 8%) [40,62,85], medication adherence [90], sexual health promotion [45], health screening [39], health education (n=2, 6%) [42,38] and communication support [97].

Several methods were used to develop the new frameworks including multidisciplinary collaboration [26,45,96], team experience after developing mHealth interventions [25,99], literature reviews [27,28], adaptation of known frameworks by themselves [91], or associated with stakeholders inputs [87], while the methods were not described in five frameworks [81,84,97,100,38]. Studies using existing frameworks reported the use of one (n=10, 28%), or the combination of more than one framework (n=12, 33%). The aim of the included frameworks varied: 26 frameworks (72%) provided guidelines for the design, development and evaluation of mHealth interventions [25,26,27,36,38,39,40,42,43,44,45,46,62,81,82,83,84,85,90,92,93,96,97,99,100,110], five frameworks (14%) focused on intervention design and development [41,89,94,95,98], one framework (3%) focused on the intervention content design [87], whilst another focused on intervention development [93]. One framework (3%) described the development and evaluation of an AI CA [28], another framework included intervention design, development, evaluation and implementation [91], and a last framework focused on platform design to evaluate app engagement [88].

Figure 2 outlines the key steps identified in mHealth frameworks. First, the intervention and target population should be clearly defined using literature reviews or engaging with key stakeholders, followed by the digital intervention design, and the development of an initial prototype using iterative, multidisciplinary, user-centered design frameworks. Once a viable prototype is available, the final steps include evaluating the feasibility of the interventions and assessing their effectiveness.


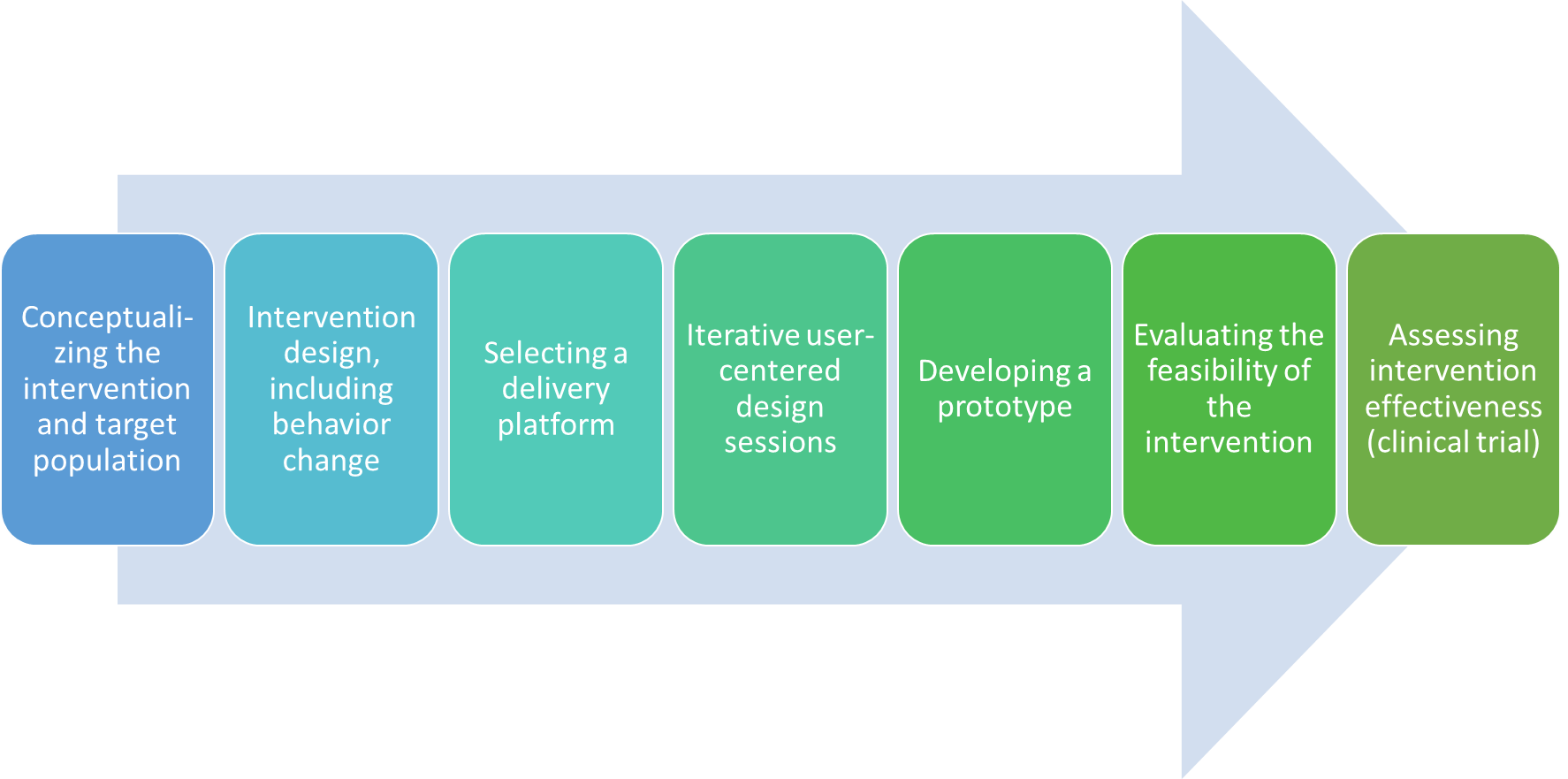


**Figure 2.** Key steps prioritized in mHealth frameworks included in the literature review.

In general, the included frameworks did not include large clinical trials to assess intervention effectiveness. For example, Zhang et al. (2020) [28] conducted a rapid preliminary literature review comprising four databases to assess current evidence on the use of conversational agents for behavioral change interventions. However, other frameworks included a thorough needs assessment involving the target population, by using one of several qualitative methods, commonly focus group discussions [25,89] or in-depth interviews [89]. The selection of the delivery platform would depend on the intervention type and data capture requirements, and included the use of phone sensor data as the only information source or to complement user-entered information [81]. End-user participation in the design process was the preferred intervention design modality, including design thinking [98] and other user-centered design techniques [85,88,95]. Once an initial prototype was completed, pilot testing of the usability, feasibility, and acceptability of the intervention may identify required system refinements [83,100] before testing it in an adequately powered, rigorous clinical trial [26].

#### CA taxonomies

Five studies reported on several classification systems for CAs. Three studies (60%) referred to all types of CAs [5,32,67], one study (20%) focused on AI CAs [65], and another study (20%) classified embodied CAs [66]. Two studies (40%) were specific for healthcare [65,66]. The focus of the classification was varied, including CA evaluation domains [65], categorization of CA design platforms [5], conversation design [67], embodied CA design features [66], and the impact of CA design on user interactions [32]. Taxonomies were based on literature reviews, alone [32,66,67] or complemented by research team experience [65] or analysis of design platforms [5]. Despite their differing focus, most taxonomies included several features that appear essential to define CAs, such as the communication modality (text- or speech-based) [5,32,65,66,67], conversation design (rule-based or AI-based) [5,32,65,67], context (either domain-specific or general purpose) [5,65,67], and focus on user data protection, particularly in healthcare-focused CAs [32,65,66]. Other features were specifically aligned to the taxonomy focus, like user engagement features such as empathy [32,66] and emotions [66], trust and credibility [32], CA looks, and degree of “humanness” [66].
