# Supplementary material for: Designing, Developing, Evaluating, and Implementing a Smartphone-Delivered, Rule-Based Conversational Agent (DISCOVER): Development of a Conceptual Framework
Source: JMIR Mhealth Uhealth. 2022 Oct 4;10(10):e38740. doi: 10.2196/38740 (PMC9579935; doi:10.2196/38740)
Supplement: Multimedia Appendix 2 [file mhealth_v10i10e38740_app2.docx]

**Multimedia Appendix 2**

Literature review of smartphone-delivered, rule-based conversational agents

**Methods**

Our group has previously conducted and published a scoping review on conversational agents in healthcare [1], using a broad literature search consisting of 63 search terms associated with “conversational agent”, (please refer to Multimedia Appendix 2). The findings of this scoping review are reported elsewhere [1].

For the current study, we conducted a new search in PubMed in December 2020 using the same search strategy. Studies were included if they reported on the development and design of rule-based, text-based CAs delivered via a smartphone, with or without a stationary visual avatar. Studies were excluded if they described speech-based or embodied CAs, used AI algorithms to design the conversations, such as IBM Watson or Watson assistant, Siri or Google assistant, included a diagnostic or symptom checker, questionnaire, or online form that didn’t engage in conversation with the user, or the studies discussed technical development with no information on the design or development of content. Embodied CAs were defined as computer-generated characters able to engage in verbal and non-verbal communication with the user [59].

The search, screening, and data extraction were performed in parallel by two reviewers (DD and LM). Any discrepancies were resolved through discussion and consensus. Data were extracted and analyzed using qualitative thematic analysis [60].

**Results**

The studies included in a previous scoping review [1] were screened for eligibility, yielding 10 studies fulfilling the criteria for inclusion in this review. The subsequent PubMed search retrieved seven additional articles, while another study was retrieved through manual search. In total, 18 papers, reporting on 14 studies were included in the review (Figure S2). Multimedia Appendix 8 presents a summary of studies describing the CA interventions.

**Figure S2.** Selection flowchart for clinical trials of CA interventions

Most of the included studies were conducted in Europe (n=11, 79%), including Switzerland (n=6, 43%) [31,33,34,72,73,74,76,77,78,79], Italy (n=2, 14%) [69,80], France (n=1, 7%) [30], Spain (n=1, 7%) [68] and Sweden (n=1, 7%) [3]. The remaining three studies were conducted in USA (n=1, 7%), Hong Kong (n=1, 7%), and Japan (n=1, 7%). The studies reported on CA interventions addressing a variety of healthcare topics. Five studies (36%) reported on healthy lifestyle changes aimed at improving diet quality in the general population [77,80], weight reduction in overweight and obese adolescents [31,33,34,72,74,79], and smoking cessation [75]. Four studies (29%) aimed at improving mental wellbeing and reducing stress in the general population [3,69,71], or young people recovering from cancer [70]. Other four studies (29%) focused on chronic disease self-management, including asthma [73], breast cancer [30], chronic pain [78], and hypertension [68]. Finally, one study (7%) presented a questionnaire for individuals receiving music therapy [76]. Half of included studies (n=7, 50%) reported a randomized controlled trial (RCT) [3,31,33,34,68,71,72,74,75,79,80], four studies (29%) reported a pilot study [69,70,77,78], while three studies reported a prospective observational study (n=1, 7%) [30], a multisite, single arm feasibility study (n=1, 7%) [73], and a usability study (n=1, 7%) [76].

In seven studies (50%), CAs were delivered through existing messaging platforms such as Facebook Messenger [30,70,77,80], Telegram [68], LINE [71], and WeChat [75], six studies used apps specially designed for the study [3,31,33,34,72,73,74,76,78,79], and one study included a web-based CA [69]. In four studies [31,33,34,72,73,74,78,79], the standalone app was developed using MobileCoach, an open-source platform for the design and delivery of CA-led digital health interventions [35]. In all but two studies that used only text [3,80], the CA used multimodal communication modalities including text, emojis, images, and video, to converse with the participants. The CA personality was briefly described in eight studies [3,33,69,70,72,73,74,76,77,78], generally as empathetic, supportive, friendly, and peer-like. One CA was described as non-judgmental [70]. At the same time, ten CAs had a name, of which four CAs had a non-gendered name [3,68,69,70], two CAs had distinctive female names [76,78], and three CAs described in four studies had female and male names and representations [31,33,34,69,72,73,74,79]. Engagement features were mentioned in 11 studies, including notifications, reminders, personalized information, gamified elements, and daily check-ins.
