# Supplementary material for: Designing, Developing, Evaluating, and Implementing a Smartphone-Delivered, Rule-Based Conversational Agent (DISCOVER): Development of a Conceptual Framework
Source: JMIR Mhealth Uhealth. 2022 Oct 4;10(10):e38740. doi: 10.2196/38740 (PMC9579935; doi:10.2196/38740)
Supplement: Multimedia Appendix 4 [file mhealth_v10i10e38740_app4.docx]

**Multimedia Appendix 4.** Methodology implemented for conceptual framework development using the conceptual framework development steps as described by Jabareen (2009).

| **Steps for Conceptual Framework development** | **Methods implemented for this framework** |
| --- | --- |
| 1. Scoping the multidisciplinary literature on the area of focus | Conducted two literature reviews:   1. scoping mHealth frameworks, 2. identifying CA design and development studies for multiple usage applications in healthcare |
| 2. Organizing the data into important categories | Categorized data into the three main categories in line with the aims of this conceptual framework:   1. design, 2. development 3. evaluation |
| 3. Naming the emerging categories and themes | Defining the goal; Creating an identity; Acquiring knowledge; Conversation flow and content building; Tone; Language; Purpose; Information; Direction; Emojis; Manpower; User experience; Behavior change theories; Timing and frequency; Hybrid; Publishing the CA; Testing the CA; Evaluate; Monitor and improve |
| 4. Breaking down the concepts and re-categorizing them | **Phase 1** - Defining the goal; Creating an identity; Acquiring knowledge,  **Phase 2** - Conversation flow and content building; Tone; Language; Purpose; Information; Direction; Emojis; Manpower; User experience; Timing and frequency  **Phase 3** - Publishing the CA; Testing the CA; Evaluate; Monitor and improve.  **Additional considerations** – Hybrid; Behavior change theories |
| 5. Condensing similar concepts  6. Synthesis, and re-synthesis for coherence | “Defining the goal” and “Creating an identity” into - **Design**  “Developing the evidence-based content”; “Conversation building” and “Conversation flow” all condensed into – **Development** (as opposed to three separate steps in the framework)  “Timing”, “frequency”; “Tone”; “Language” condensed into – **Development** >> Building the conversation flow  “Piloting”; “Monitoring”; “Evaluating”; “Improving” condensed into – **Evaluation and implementation**.  Co-design initially placed in phase 1 and then removed as possible a floating theme. |
| 7. Validating the conceptual framework | 1. Conducted a validated study involving the development of healthy lifestyle change CA “Precilla” which provided an opportunity to test the preliminary framework and identify areas for improvement. 2. Presentation of framework at conferences and meetings with colleagues. |
| 8. Reworking the framework based on feedback and new insights | Concepts and themes reorganized and optimized based on feedback from the validation study and feedback from colleagues. Finalized framework (DISCOVER) presented in this paper |
