# Supplementary material for: Designing, Developing, Evaluating, and Implementing a Smartphone-Delivered, Rule-Based Conversational Agent (DISCOVER): Development of a Conceptual Framework
Source: JMIR Mhealth Uhealth. 2022 Oct 4;10(10):e38740. doi: 10.2196/38740 (PMC9579935; doi:10.2196/38740)
Supplement: Multimedia Appendix 5 [file mhealth_v10i10e38740_app5.docx]

**Multimedia Appendix 5.** Mapping the steps of the conceptual framework applied to the design, development, and evaluation of Precilla.

**Methods**

The resulting framework was used to develop a rule-based, text-based, smartphone-delivered CA prototype, designed to support healthy lifestyle change and educate participants about diabetes. The CA (*Precilla*) delivered advice on healthy living over four weeks with a focus on healthy eating, physical activity, stress management, and healthy sleeping habits. We tested the feasibility and acceptability of using *Precilla* for healthy lifestyle behavior change in a web-based, single-arm feasibility study[63,64]. The study was approved by the Nanyang Technological University Institutional Review Board (IRB-2018-11-032).

**Results**

The conceptual framework was used to develop a CA offering a healthy lifestyle behavior change intervention consisting of educational information on diabetes and prediabetes and advice to support healthy lifestyle changes. We used the online platform *“Chatfuel”* to create a low-cost, text-based, rule-based CA hosted by Facebook Messenger. The design and development steps closely followed the conceptual framework steps as outlined in the Table below.

| **Conceptual framework steps** | | **Implementation for Precilla** |
| --- | --- | --- |
| **Design** | **Defining the goal** | |
|  | Needs Assessment/Co-design | Literature review and semi-structured interviews [63] to assess:  - knowledge on diabetes and prediabetes  - views on healthy living  - barriers and facilitators to a healthy lifestyle |
|  | The aim | To inform and educate the general population in Singapore on healthy lifestyle changes (diet, physical activity, sleep, and stress management). |
|  | Defining the end-user | General population in Singapore  Individuals interested in the use of digital health for healthy lifestyle change. |
|  | **Creating the CA identity** | |
|  | CA personality | Female healthcare coach-like named Precilla  Formal yet friendly  Human-like cartoon avatar |
|  | Tone and language | English  Simple, direct language |
|  | **Selecting the delivery interface** | |
|  | Delivery Channel | Facebook Messenger |
|  | Communication modalities | Text, Images, Videos |
|  | **Assembling a multidisciplinary team** | |
|  | - Assistant Professor of Evidence-Based Medicine and Digital Health (MD, PhD Public Health) - Team leader  - Post-doctoral researcher (MBBS, MSc, PhD)  - Research Associate (BSc, MSc in Public Health)  - PhD Student (BSc) | |
| **Development** | **Developing the content** | |
|  | Evidence-based information | - Reputable health websites (Health Promotion Board, Singapore)  - Peer-reviewed literature on sleep and stress management |
|  | Types of messages | - Education on pre-diabetes  - Advice on:  - Weekly duration and intensity of exercise  - Methods to improve sleep and manage stress  - Healthy diet |
|  | Behavior change theories | Capability, Opportunity, Motivation, Behavior (COM-B) model of behavior change |
|  | **Building the conversation flow** | |
|  | Providing suitable answer options | Up to 3-4 button options |
|  | Selecting a mapping tool | X-mind mind mapping software |
|  | Selecting appropriate message timing and frequency | Messages to be sent 4 times a week (Mon, Weds, Fri, Sun) at Noon (lunchtime). |
|  | Employing engagement strategies | Facebook Messenger sent notifications whenever a new interaction is due. |
| **Evaluation and Implementation** | **Efficacy and effectiveness of the CA intervention** | |
|  | Pilot study testing feasibility and acceptability | |
|  | **User engagement and acceptability** | |
|  | Qualitative and quantitative measures | |

The needs assessment consisted of a literature review and qualitative, semi-structured interviews with 20 adult individuals from Singapore, fluent in English, that owned a Facebook account, who were not pregnant or had a severe chronic condition [63]. Participants offered their perceptions of CAs and smartphones in Singapore, discussed their knowledge about diabetes and prediabetes, and their views on a healthy lifestyle.

The feasibility and acceptability of the CA were evaluated in a web-based single-arm pilot feasibility study including 75 participants who received messages from the CA four times a week, for four weeks [64]. We assessed the feasibility of participants’ recruitment and retention, the usability of the CA, participants’ satisfaction, and clinical outcomes including quality of life (QoL), diabetes knowledge and risk perception, and self-reported diet, exercise, sleep, and stress measures. The assessments included self-reported questionnaires and data analytics provided by the Chatfuel platform. The study demonstrated that the delivery of healthy lifestyle advice using a CA hosted in Facebook Messenger was feasible and acceptable, although our recruitment strategy was unable to enroll the target sample size. Participant retention and engagement were high.

We also conducted semi-structured interviews with a subset of 20 participants (manuscript currently under review). The interviews aimed to understand the participants’ views on Precilla, its acceptability, participants’ satisfaction with the intervention, as well as suggestions for improvement in the future. Most participants valued Precilla’s friendly personality and informative content that motivated some positive changes to their lifestyle. Additionally, other participants considered the content too simple, and the messages lengthy with insufficient answer options. Participants favored personalized content and suggested future versions may include more information on the clinical presentation of diabetes and prediabetes, specific healthy food recommendations, and different levels of intensity for suggested exercises. They also suggested the inclusion of more diverse answer options for the user to choose from.
