# Supplementary material for: Designing, Developing, Evaluating, and Implementing a Smartphone-Delivered, Rule-Based Conversational Agent (DISCOVER): Development of a Conceptual Framework
Source: JMIR Mhealth Uhealth. 2022 Oct 4;10(10):e38740. doi: 10.2196/38740 (PMC9579935; doi:10.2196/38740)
Supplement: Multimedia Appendix 6 [file mhealth_v10i10e38740_app6.docx]

**Multimedia Appendix 6:** Design, development, and evaluation frameworks for mHealth interventions

| **Study ID [REF]**  **Country** | **Area of healthcare** | **mHealth intervention** | **Name of the framework(s) [REF]** | **New or existing** | **Development methodology** | **Framework focus** | **Number of steps** | **Conceptual framework features** |
| --- | --- | --- | --- | --- | --- | --- | --- | --- |
| Bauerle Bass 2018 [36]  USA | Injection drug users treated with methadone who are Hepatitis C+ | mHealth tool to provide health information about Hepatitis C | Formative evaluation framework | Unclear | Not mentioned | Design, development, and evaluation | 6 | 1- Perceptual mapping surveys  2- Message strategy development (map the spatial relationship of HCV treatment constructs and develop message strategies)  3- Message strategy and mood board development  4- Concept testing  5- Final tool development (engaged a private developer)  6- User testing with HCV + methadone patients |
| Chen 2019 [37]  China | Behavior change applied to the promotion of healthy diet and physical activity in patients with coronary heart disease | mHealth intervention + smartphone app (iCARE) | IM [37] | Existing framework | Application of existing framework | Intervention development | 3 | 1- **Needs assessment**: a- Literature review  b- In-depth interviews and focus group discussions   with coronary heart disease patients  2- **Development of a matrix of objectives** for diet and physical activity change according to the CAM Model  3- **Formulation of evidence-based methods** and strategies, and practical applications:   a- Define general and desirable criteria to select   evidence-based methods  b- Literature review of existing evidence-based   methods and strategies  c- Team discussions and expert consultations to   select effective evidence-based methods and   strategies  d- Translate evidence-based methods into   practical applications  e- Formulate the final list of interventions to use   in iCARE |
| Curtis 2015 [38]  UK | Parents of children with overweight to support healthy eating | Smartphone app | UCD [38]  BCW [39] | Existing framework | Combination of existing frameworks | Design and development | 10 | **Understanding the Problem and User Preferences**: 1- Defining the Problem (literature review)  2- Selecting the Target Behavior (user and expert involvement)  3- Specify the Target Behavior  4- Understanding the Target Behavior and User Preferences (qualitative research (e.g. focus groups) with end-users and experts)  **Translating Research Findings Into App Features**: 5- Select User Preferences, according to relevance to the target behavior, availability online, ease of implementation, alignment with usability and UX recommendations, and evidence-based  6- Select Intervention Functions, according to COM-B  7- Select Behavior Change Techniques  8- Translate Behavior Change Techniques Into App Features, in consultation with app developers  **Pre-Testing and Refinement**: 9- Piloting Potential App Features, through focus groups with users  10- Refining App Features, Generating Content, and Developing the Prototype |
| Depp 2016 [81]  USA | Bipolar disorder  Early detection of mood episodes | Smartphone apps  Text messaging  Home-based telehealth  Wearable sensors | Not mentioned | New framework | Not mentioned | Design, development, and evaluation | 5 | 1- Platform considerations  2- Inputs and Outcomes (sensor data, PROs)  3- Application of Prediction Models  4- Creation of Decision Rules  5- Feedback and Clinical Application |
| Direito 2018 [82]  New Zealand | Physical Activity Promotion  Behavior change | Smartphone app | IM [37]  BIT [16] | Existing frameworks | Adaptation of existing frameworks | Design, development, and evaluation | **IM**: 3 initial steps  **BIT**: 4 steps | **IM**: 1- Needs assessment of the target population  2- State change objectives and logic model of change  3- Theory-based development of the program  **BIT**: 1- Profiler (defines the user and environment)  2- Intervention planner  3- Intervention repository  4- User interface |
| Duff 2018 [83]  Ireland | Rehabilitation exercise for cardiovascular disease | Smartphone app | mHealth Development and Evaluation Framework | Unclear | Not mentioned | Design, development, and evaluation | 4 steps in 2 phases | **Phase 1 - Conceptualization:** 1- Systematic review  2- Consultation with advisory panel  **Phase 2 - Usability and Acceptability Testing: 3-** Focus groups 4- Feasibility test |
| Farao 2020 [39]  South Africa | Latent tuberculosis screening | Smartphone app | ISR [40]  Design thinking [41] | Existing frameworks | Combination of existing frameworks | Design, development, and evaluation | 5 steps in 3 phases | 1- **Relevance cycle**, with a- Empathize mode; and b- Define mode. Captures the information gathered from end-users and the end-user environment  2- **Design cycle**, with a- Ideate mode; and b- Prototype mode. Useful to developing a usable prototype.  3- **Rigor cycle**, with a- Test mode. Divided in 2 phases: literature review to inform the relevance and design cycles; and testing the initial prototype |
| Fedele 2019 [84]  USA | Pediatric psychology  Behavior change | Smartphone apps  Other | IDEAS framework [18] | Existing framework | NA | Design, development, and evaluation | 10 steps grouped in 4 categories | (See Mummah 2016 for framework outline). Four integrated categories: Integrate, Design, Assess, and Share |
| Fjeldsoe 2012 [100]  Australia | Behavior change applied to physical activity in mums of young children | SMS | mHealth Development and Evaluation Framework described by Whittaker et al. [31] | Existing framework | NA | Design, development, and evaluation | 5 | 1- Conceptualization (literature review)  2- Formative research (focus groups)  3- Pre-testing, intervention development and feasibility and acceptability assessment  4- Pilot testing (pilot RCT)  5- Qualitative evaluation for refinement of the intervention with a variety of stakeholders |
| Good 2019 [85]  UK | Improving healthcare delivery to rural areas; providing advice to pregnant women via SMS | SMS | Activity theory  UCD [38] | Existing frameworks | Combination of existing frameworks | Design, development, and evaluation | 4 | 1- Understand and specify the context of use  2- Establish the user and/ or organizational requirements  3- Produce design solutions  4- Evaluate designs against requirements. |
| Jennings 2019 [87]  Bangladesh | Diabetes prevention and management  Behavior change | Voice messages | Not mentioned | New framework | -Semistructured interviews and focus group discussions  -Based on existing frameworks: COM-B model of behavior change [42] and  TDF [43] | Content design | 6 | 1- Define the context of the intervention  2- Define outcomes  3- Identify enablers and barriers to behavior change  4- Categorize the barriers and enablers according to COM-B and TDF  5- Suggest behavior change approaches for each enabler and barrier  6- Produce table of message content based on intended outcomes, barriers and enablers, and BCTs to guide scriptwriters |
| Jindal 2018 [40]  India | Diabetes and hypertension management CDSS for healthcare providers in low-resource settings | Tablet-computer mHealth intervention | UK's Medical Research Council (MRC) framework for complex interventions [44] | Existing framework | Application of existing framework | Design, development, and evaluation | 4 | 1- Identifying gaps in usual care, literature review and situation analysis of healthcare facilities  2- Identifying the components of the intervention, consultation workshops and meeting with key stakeholders  3- Developing intervention components, including development of clinical algorithms and the mHealth application  4- Evaluating acceptability and feasibility, through pilot testing |
| Kazemi 2018 [41]  USA | Binge drinking in college students | Smartphone app (REMIT) | Adaptation of IDEAS [18] | Existing framework | Adapted IDEAS framework, further informed by EMI, MI and TTM | Design and development | 5 | 1- Understand the users (literature review and focus groups)  2- Determine target behavior  3- Intervention based in behavioral theory (EMIs, MI, TTM)  4- Create delivery strategies  5- Develop the REMIT prototype |
| McBride 2018 [42]  Canada/Vietnam | Maternal and infant health knowledge, and behavior in women living in remote areas of Vietnam | SMS | Modified framework based on: 1- WHO framework and standards for country health information systems; 2- Philbrick strategic framework on mHealth for MNCH; 3- Mobile Alliance for Maternal Health Global Monitoring and Evaluation Framework, and 4- WHO mHealth framework for assessing MNCH mHealth intervention impact on health systems | Existing frameworks | Integration of existing frameworks | Design, development, and evaluation | 4 | 1- **Development** of an mHealth platform integrated into the existing health management information system  2- **Ethnographic fieldwork** (focus groups, in-depth interviews, and literature review) and **Intervention content development**, based on well-known maternal care frameworks  3- **Intervention piloting and implementation**  4- **Evaluation** of the intervention’s impact, through initial ethnographic fieldwork, pre- and post-intervention surveys, regular monitoring and evaluation visits, and mid-term and final evaluations |
| Modi 2015 [62]  India | Maternal, newborn, and child health services provided by community health workers | Smartphone app for health workers + website for medical officers (ImTeCHO) | UK's Medical Research Council (MRC) framework for complex interventions [44] | Existing framework | Not mentioned | Design, development, and evaluation | 4 | 1- Identify gaps in usual care (literature review and expert consultation)  2- Design intervention components and model their impact, using UCD principles in consultation with experts  3- Evaluate acceptability and feasibility of intervention components, relevant stakeholders interviews  4- Pilot operational delivery of the intervention |
| Mohr 2014 [27]  USA | Behavior change applied to physical or mental health, or wellness | Smartphone apps  Sensors  Web-based | Behavioral Intervention Technology Model (BIT and BIT-Tech) | New framework | Literature review | Design, development, and evaluation | **BIT**: 5 steps  **BIT-Tech**: 4 steps | **BIT**: 1- “Why”: Intervention aims  2- “How” (conceptual): Behavioral intervention strategies  3- “What”: BIT elements  4- “How (technical)”: Characteristics  5- “When”: Workflow  **BIT-Tech**: 1- Profiler, defines the user and environment  2- Intervention planner, for current interventions  3- Intervention repository, to store all intervention elements  4- User interface |
| Morton 2015 [110]  UK | Physical activity promotion for people at high-risk of type 2 diabetes | SMS & Pedometers (PROPELS) | Modified framework based on Dijkstra and De Vries model [45] for development of computer-generated interventions and evaluation framework by Whittaker [31] and Fjeldsoe [9] | Existing framework | Not mentioned | Design, development, and evaluation | 4 | 1- Conceptualization (focused literature review to define intervention objectives and BCTs)  2- Formative research (ethnography and focus groups)  3- Pre-testing ("think aloud" focus groups to explore views to text messages and intervention)  4- Piloting the intervention, to resolve technical issues. Follow-up interviews to assess acceptability and feasibility of the intervention) |
| Mummah 2016 [26]  USA | Behavior change applied to the promotion of vegetable consumption in overweight adults | Smartphone app | IDEAS framework | New framework | Multisectoral collaboration between researchers, designers, and engineers | Design, development, and evaluation | 10 steps, grouped in 4 categories | **Integrate**: 1- Empathize with target users  2- Specify target behavior  3- Ground in behavioral theory  **Design**: 4- Ideate implementation strategies  5- Prototype potential products  6- Gather user feedback  7- Build minimum viable product  **Assess**: 8- Pilot efficacy and usability  9- Evaluate efficacy in RCT  **Share**: 10- Share intervention & findings |
| Patel 2017 [43]  USA | Development of mHealth apps | Smartphone apps | BUS Framework | New framework | Adaptation of existing frameworks (BCTs, UCD, Social marketing) | Design, development, and evaluation | 6 | 1- Situational analysis, determine health problem and behavioral determinants, target users, key stakeholders  2- Concept generation and prototype design, determine the intervention focus by engaging target users (focus groups, interviews, surveys)  3- Prototype development, iterative with frequent user inputs  4- Pilot testing in a sample of end-users  5- Campaign launch (or intervention deployment), with the support of relevant stakeholders  6- Evaluation, in three aspects: process, outcome, and impact |
| Pham 2018 [88]  Canada | Self-management of chronic pain in young people | Smartphone app | UCD [38] | Existing framework | Not mentioned | Design platform to define, operationalize and evaluate effective app engagement | 3 | 1- Concept generation and ideation  2- Prototype design and system development  3- Evaluation |
| Ravn Jakobsen 2018 [89]  Denmark | Self-management of osteoporosis | Smartphone app | Participatory design process | Existing framework | Not mentioned | Design and development | 3 | 1- Identification of users’ needs (literature review, interviews, focus groups)  2- Iterative design and development of prototypes (workshops with key stakeholders and prototype testing)  3- Test mHealth app (evaluation and re-testing). |
| Schellong 2019 [91]  Germany | Post-traumatic stress disorder (PTSD) | Smartphone app | Not mentioned | New framework | Based on international standards requirements and ISR method [40], and adapted for PTSD | Design, development, evaluation, implementation | 12 | 1- Define healthcare goals informed by patient needs  2- Establish appropriate IT architecture, including interoperability  3- App functionalities and layout (PTSD specific)  4- Apply medical norms (PTSD specific), by complying with evidence-based guidelines  5- Apply technical norms (open standards, data privacy and security, etc.)  6- Importance of interdisciplinary communication  7- Use proven methods of design (user-centered design, 5-cycle model)  8- Alpha and beta test with and without patients  9- Evaluation and assessment, before release  10- Release app  11- Localization of app to specific country settings  12- Post-market communication and maintenance |
| Schnall 2016 [92]  USA | HIV prevention for high-risk MSM | Smartphone app | Adaptation of the ISR framework [40] for mHealth apps | Existing framework | Not mentioned | Design, development, and evaluation | 3 cycles | 1- **Relevance cycle**, understand the end-user environment and requirements (focus groups)  2- **Rigor cycle**, or knowledge base (literature review and apps assessments)  3- **Design cycle**: i. Design session 1: identify features and content  ii. Design session 2: user interface and design prototypes  iii. Evaluation, of the prototypes (experts and end-users) |
| Skrabal Ross 2019 [90]  Australia | Improve adherence to oral chemotherapy in adolescent and adult cancer patients | SMS | BIT [16]  mHealth Development and Evaluation Framework described by Whittaker et al. [31] | Existing frameworks | Combination of existing frameworks | Design, development, and evaluation | 3 | 1- **Conceptualization of the program**, literature and scoping review, identification of relevant behavioral change strategies  2- **Definition of (technical) features and structure of the program**, needs assessment with end-users  3- **Selection of program delivery technology**, depending on availability and relevance to target population |
| Sporrel 2020 [44]  The Netherlands | Physical Activity Promotion  Behavior change | Smartphone app (PAUL) | IDEAS [18]  BIT [16] | Existing frameworks | Adaptation of existing frameworks (IDEAS, BIT, COM-B & FBM) | Design, development, and evaluation | 9 | **Phase 1 (Preliminary work)**: 1- Identify target behavior and study aims (consultation with stakeholders and interviews with end-users)  2- Identify barriers for performing physical activity  3- Design and pilot initial idea, based on stakeholders input  **Phase 2**: 4- Theoretical grounding to develop a conceptual model based on BCTs and literature reviews  5- Identify requirements with end-users (focus groups)  6- Design functional components, based on BIT  7- Technical operationalization  8- Feasibility testing  9- Effect study |
| Sun 2020 [45]  USA | Sexual health promotion and HIV prevention for transgender women | Smartphone app (Trans Women Connected ) | Not mentioned | Unclear | Not mentioned | Design, development, and evaluation | 3 | 1- Formative research (focus groups)  2- Prototype design and development (curriculum and storyboards and wireframes development, in consultation with relevant stakeholders)  3- Usability testing ("think aloud" method) |
| Tay 2017 [93]  Australia | Behavior change applied to self-monitoring of calcium intake | Smartphone app | Not mentioned | Unclear | Not mentioned | Design, development, and evaluation | 4 | 1- Conceptualization  2- Iterative development and pretesting with end users  3- Pilot testing  4- Mixed methods evaluation (questionnaires, focus groups) |
| Van Agteren 2018 [94]  Australia | Smoking cessation  Behavior change | Smartphone app | IM [37]  PSD [46]  *Content*: TDF [43] & BCTs for smoking cessation [47] | Existing framework | Not mentioned | Design and development | 6 | **IM**: 1- Needs assessment of health behavior  2- Definition of program objectives  3- Selecting evidence-based methods and practical applications  4- Development of the mHealth app  5 & 6- Adoption, implementation, and evaluation |
| Verbiest 2019 [46] New Zealand/The Netherlands | Behavior change intervention for indigenous communities focusing on physical activity, healthy eating and family | Smartphone app (OL@-OR@) | Participatory codesign cycle [48]  TDF [43] | Existing framework | Adaptation of existing frameworks | Design and development | 6 & 4  (both design activities occurred in parallel) | **Participatory codesign cycle**: 1- Opportunity identification (consultation with stakeholders)  2- Knowledge generation (focus groups with end users to understand important aspects of health and wellbeing)  3- Elucidation of needs and desires, regarding the mHealth tool (focus groups)  4- Description of the mHealth requirements  5- Envisaging the mHealth tool  6- Prototype testing, iterative manner  **Content development of behavior change intervention**: 1- Identification of key content modules  2- Identification of relevant determinants of behavior change  3- Selection of appropriate BCTs  4- Incorporating BCTs in mHealth intervention |
| Vilardaga 2018 [95]  USA | Smoking cessation for people with serious mental illness | Smartphone app | UCD [38] | Existing framework | Adaptation of UCD | Design and development | 7 | 1- Understanding and defining the context of use, expert panels  2- Selecting design principles  3- Incorporating evidence-based smoking cessation content  4- Ideation, sketching and paper prototyping  5- Usability testing of paper prototype  6- Defining app vision and design specifications  7- Engaging software development vendor |
| Whittaker 2012 [25]  New Zealand | Smoking cessation Depression | SMS  MMS | Not mentioned | New framework | Informed from team experience developing 3 mHealth interventions | Design, development, and evaluation | 6 steps and 3 overarching themes | **Steps**: 1- Formative research (inform content)  2- Pretesting (determine acceptability and refine) 3- Pilot study (test content and processes)  4- RCT (test effectiveness)  5- Qualitative research (intervention improvement)  6- Evaluation of intervention impact  **Overarching themes**: 1- Involve target population  2- Theoretical basis (intervention mapping)  3- Implementation focus |
| Wilhide III 2016 [96]  USA | Chronic disorders | Smartphone app | Chronic Disease mHealth App Intervention Design Framework | New framework | Multidisciplinary expert collaboration informed by the CCM [49], evidence-based clinical and behavioral programs and health care outcomes | Design, development, and evaluation | 7 | **Strategic plan**: 1- Identify value drivers (or aims of the app)  2- Define outcome measures of interest  3- Identify program goals for key stakeholders (users)  **Intervention plan**: 4- Behavior domains (essential behaviors, supporting actions and determinants)  5- Multidimensional profile (personalization and intervention customization)  6- Evidence-based clinical and behavioral interventions  7- App features and content. |
| Wittenberg 2019 [97]  USA | Communication support to cancer caregivers | Smartphone app | Not mentioned | New framework | Not mentioned | Design, development, and evaluation | 8 | 1- Review of existing print resources  2- Select theoretical framework for content development  3- Integrate stakeholder feedback and literacy assessment of target population  4- Review of existing mHealth resources  5- Prototype development  6- Assess caregiver acceptability of the prototype  7- Provider assessment of quality and perceived impact  8- Caregiver acceptability of final product |
| Woods 2018 [98]  Australia | Heart failure self-management | Smartphone app | ISR framework [40]  Design Thinking Process [41] | Existing framework | Adaptation of ISR for healthcare context | Design and development | 3 (ISR)  5 (Design Thinking) | **ISR**: 1- Relevance cycle  2- Rigor cycle  3- Design cycle  **Design Thinking Process**: 1- Empathize (needs assessment with patients and their carers)  2- Define, the healthcare problem and list potential opportunities  3- Ideate workshops for ideas generation  4- Prototype based on the design best ideas  5- Test, using validated tools to assess usability |
| Yardley 2015 [99]  UK | Behavior change | Smartphone app | Person-based approach | New framework | Practical experience of a research team that developed several eHealth interventions | Design, development, and evaluation | 4 | 1- Planning (months 0-6)  2- Design (months 3-9)  3 Development and evaluation of acceptability and feasibility (months 6-18)  4- Implementation and trialing (months 12-18) |
| Zhang 2020 [28]  USA | Physical activity and healthy diet promotion | AI CA | AI Chatbot Behavior Change Model | New framework | Literature review | Development, and evaluation | 4 | 1- Designing chatbot characteristics and understanding user background  2- Building relational capacity (e.g. social dialog, empathy, humor, self-disclosure, etc.)  3- Building persuasive conversational capacity, using evidence-based behavioral change theories and techniques, EMAs, sensor data, JTAIs, etc.  4- Evaluating mechanisms and outcomes, including user experience, usage pattern, conversational quality, relational capacity, and behavioral outcomes |

**AI**: Artificial Intelligence; **BCT**: Behavior Change Theory; **BCW**: Behavior Change Wheel; **BIT**: Behavioral Intervention Technology; **CA**: Conversational Agent; **CAM**: Contemplation-Action-Maintenance Model; **CCM**: Chronic Care Model; **CDSS**: Clinical Decision Support System; **COM-B**: Capability, Opportunity, Motivation, Behavior; **EMA**: Ecological Momentary Assessment; **EMI**: Ecological Momentary Interventions; **FBM**: Fogg’s Behavior Model; **IM**: Intervention Mapping; **iCARE**: **I**ndividualized, **I**ntelligent and **I**ntegrated **C**ardiovascular **A**pplication for **R**isk **E**limination; **ISR**: Information Systems Research; **JTAI**: Just-In-Time Adaptive Interventions; **MI**: Motivational Interviewing; **MMS**: Multimedia Messaging Service; **MNCH**: Maternal, Newborn and Child Health; **MSM**: Men who have sex with men; **NIATx**: Network for the Improvement of Addiction Treatment; **PAUL**: Playful data-driven Active Urban Living; **PRO**: Patient Reported Outcomes; **PROPELS**: Promotion Of Physical activity through structured Education with differing Levels of ongoing Support for those at high risk of type 2 diabetes; **PSD**: Persuasive Systems Design; **RCT**: Randomized Controlled Trial; **REMIT**: Reductions through Ecological Momentary/ Motivational Intervention/Transtheoretical; **SMS**: Short Message System; **TDF**: Transtheoretical Domains Framework; **TTM**: Transtheoretical Model; **UCD**: User-Centered Design; **UX**: user experience; **WHO**: World Health Organization
