# Supplementary material for: Designing, Developing, Evaluating, and Implementing a Smartphone-Delivered, Rule-Based Conversational Agent (DISCOVER): Development of a Conceptual Framework
Source: JMIR Mhealth Uhealth. 2022 Oct 4;10(10):e38740. doi: 10.2196/38740 (PMC9579935; doi:10.2196/38740)
Supplement: Multimedia Appendix 7 [file mhealth_v10i10e38740_app7.docx]

**Multimedia Appendix 7**: Classification systems for conversational agents

| **Study ID [REF]**  **Country** | **Restricted to a study field?** | **Type of CAs** | **Type of taxonomy** | **Development methodology** | **Taxonomy elements** |
| --- | --- | --- | --- | --- | --- |
| Denecke 2020 [65]  Switzerland | Yes. Healthcare | AI (unrestricted natural language) | CA Evaluation | Team experience developing 4 CAs  Literature review | *CA Evaluation domains*  1- Task-oriented perspective (task completion/ task success/ result quality/ data provenance)  2- Artificial intelligence perspective (human-like/ emotional connection/ recognizing limitations/ error handling)  3- System quality perspective (data security/ privacy policy/ compliance with data management regulations/ performance)  4- Linguistic perspective (conversation effectiveness/ relevance/ context awareness/ dialogue quality/ empathy)  5- UX perspective (usability/ adherence/ user-friendliness/ user engagement and satisfaction, others)  6- Healthcare quality perspective (patient safety/ appropriateness/ health outcomes aligned with evidence-based practices) |
| Diederich 2019 [5]  Germany | No | All | Design platforms | Database of design platforms  Literature review Empirical classification of CA platforms | *Platforms classification*  1- Communication mode or user interface (text-based/ speech-based/ both)  2- Context (general purpose/ domain-specific)  3- Language (single language/multiple languages) 4- Intelligence (rule-based/ self-learning)  5- Implementation (programming/ modelling/ supervised learning/ hybrid) 6- Hosting (on premise/ cloud/ both)  7- Reporting about CA function (without reporting/ with reporting) 8- Pricing model (usage based/ user based/ instance based/ free) 9- Sentiment detection (with sentiment/ without sentiment) 10- Enterprise integration (none/ API/ pre-build interface)  11- Platform integration (single platform/ cross-platform)  *Design platforms archetypes*  1- Multi-language, integrative CA platform with advanced analytical functionality  2- General-purpose, cloud-based CA platform with single language and API support  3- Text-based, domain-specific CA platform with modeling functionality |
| Hussain 2019 [65]  Australia | No | All | Conversation design | Not mentioned | *CA classification*  1- Interaction mode (text-based/ voice or speech-based)  2- CA application (task-oriented/ non-task-oriented)  3- Rule-based or AI (machine learning/ deep-learning)  4- Domain-specific or open-domain  *Conversation design approach*  1- Rule-based  2- Retrieval-based  Examples of techniques used in Rule- and Retrieval-based CAs (parsing/ pattern matching/ chatscript/ AIML/ ontologies/ Markov chain model)  3- Generative-based (Artificial Neural Networks models – RNN, Seq2Seq, LTSM) |
| Ter Stal 2020 [66]  The Netherlands | Healthcare | Embodied CAs | Design features | Literature review | *CA output*  1- Emotional/ non-emotional agent  2- Relational, empathic behavior/ non-relational, empathic behavior  3- Disclosure of personal information/ no self-disclosure  4- Variability of agent’s emotions or behaviors/ no variability  5- User controlled prosody and facial expression/ non-user control  6- Interactivity/ non-interactivity  7- Linguistic tailoring  8- Communication text-based/ speech-based  9- Friend/ professional  *CA looks*  1- Rendering style (human-like/ cartoon-like)  2- Clothing (professional/ casual  3- Body shape (slim/ fat)  4- Gender (female/ male)  5- Age (young/ old)  6- Cultural tailoring/ no cultural tailoring |
| Zierau 2020 [32]  Switzerland | NA (not restricted to healthcare) | All | CA features impact on user interactions | Systematic review | *Perceptual and attitudinal outcomes*  1- Rapport or social bond (likeability/ engagement/ perceived closeness)  2- Social presence (humanness/ social presence)  3- Trust (credibility/ privacy perceptions)  4- Utility (usefulness/ ease of use/ interaction quality/ satisfaction/ helpfulness)  5- Behavioral intention & outcomes (intention to use/ willingness to interact/ interaction behavior/ behavior change)  *CA features*  1- Auditory (voice quality/ voice gender)  2- Interaction (pre-set answers/ natural dialog)  3- Invisible (conversation timing (chronemics)/ intelligence/ personality/ haptics)  4- Visual (agent appearance/ gender/ body movements (kinesics)/ CMC (emojis/ typos/ images/ videos))  5- Verbal or communication style (content/ contextual information/ user content) |

**AI**: Artificial Intelligence; **AIML**: Artificial Intelligence Mark-up Language; **BIT**: Behavioral Intervention Technology; **CA**: Conversational Agent; **CMC**: computer-mediated communication; **LTSM**: Long Short-Term Memory Networks; **RNN**: Recurrent Neural Network; **Seq2Seq**: Sequence to Sequence Neural Model
