# Supplementary material for: Designing, Developing, Evaluating, and Implementing a Smartphone-Delivered, Rule-Based Conversational Agent (DISCOVER): Development of a Conceptual Framework
Source: JMIR Mhealth Uhealth. 2022 Oct 4;10(10):e38740. doi: 10.2196/38740 (PMC9579935; doi:10.2196/38740)
Supplement: Multimedia Appendix 8 [file mhealth_v10i10e38740_app8.docx]

**Multimedia Appendix 8.** Characteristics of clinical trials on rule-based CAs

| **Study ID [REF]**  **Country of origin** | **Study design** | **Healthcare focus** | **CA intervention** | **CA name & personality** | **Delivery channel** | **Communication modalities** | **Engagement features** | **CA development process** | **CA Evaluation** | |
| --- | --- | --- | --- | --- | --- | --- | --- | --- | --- | --- |
|  |  |  |  |  |  |  |  |  | **N° participants/ Duration of intervention** | **Results** |
| Carfora 2019 [80]  Italy | RCT | Healthy eating | Persuasive messages to deter red and process meat consumption | Not mentioned | Facebook Messenger | Text | None | Not mentioned | 180 undergraduate students  Intervention: 2 weeks  Study: 2 months | Emotional messages are more effective than informational ones |
| Casas 2018 [77]  Switzerland | Pilot study | Healthy eating | Coaching aimed at decreasing meat or increasing fruits and vegetables consumption. | No name  Friendly, positive, and stimulating | Facebook Messenger | Text, emoticons, images, videos, others | Food diary, notifications, weekly summaries, health-related information | Development platform: Chatfuel | 36 persons  4 weeks | 11% challenges were successful  65% people improved consumption |
| Chaix 2019 [30]  France | Prospective observational study | Breast cancer | Personal health assistant that educates patients by answering questions on breast cancer. | Vik  Informative | Facebook Messenger and web-based | Text, emojis | Reminders (medication & appointments)  Daily questions | Developed by Wefight Inc.  Information modules delivered to users according to user requests | 958 patients with breast cancer and their relatives  1 year | High user satisfaction  Improved medication adherence |
| Denecke 2018 [76]  Switzerland | Usability study | Music therapy (valid for other specialties as well) | Self-Anamnesis | Ana  Human-like, funny, friendly | Standalone app | Text, speech (Android) | Use of motivational statements by the chatbot and opportunity for users to ask queries. | Development platform: Pandorabots  Conversation developed using AIML language  Content developed by literature review and expert advice | 22 adults  30-45 mins | System was “fun to use” and engaging  Participants found some elements of the system challenging to use |
| Echeazarra [68] 2021  Spain | RCT | Hypertension | Self-monitoring of blood pressure | TensioBot  Informative | Telegram | Text, image, video | Reminders to take BP and for next medical appointment.  Information on BP management | Not mentioned | 112 patients with hypertension  2 years | No difference in adherence to BP monitoring schedule  Improved knowledge and satisfaction with the intervention |
| Gabrielli 2020 [69]  Italy | Pilot study | Mental health | Life skills coaching and well-being promotion | CRI (Cristina) & CRIS (Cristiano)  Nice, smart old friend, trustworthy | Website | Text, images, video | Personalized features (CA same gender as user) | Co-designed with adolescents | 21 adolescents  4 weeks | Intervention was useful, easy to use and innovative |
| Greer 2019 [70]  USA | Pilot RCT | Mental health | Psychosocial needs of young people treated for cancer  Positive psychology | Vivibot  Non-judgmental | Facebook Messenger | Text, video, others | Not mentioned | Developed by Hopelab  User-centered design with young adults  Information prewritten and automatically delivered | 45 young adults who completed cancer treatment  Intervention: 4 weeks  Study: 8 weeks | Reduction in anxiety in experimental group, larger if more interactions with CA |
| Hauser-Ulrich 2020 [78]  Switzerland | Pilot RCT | Chronic pain | Self-management of chronic pain using psychoeducation and CBT-based pain management | SELMA  Supportive, sympathetic, empathic, with a sense of humor | Standalone app | Text, emojis, audio, video | Personalized text messages  Recollection of earlier tasks  Notifications  Overview of modules | Development platform: Mobile coach open-source software platform for the design and evaluation of mobile TBHCs  Predefined text  Preselected responses | 102 individuals with cyclical or chronic pain  8 weeks | No significant change in pain-related impairment  Intention to change behavior positively correlated with pain-related impairment and pain intensity  Users found the intervention useful and enjoyable |
| Kamita 2019 [71]  Japan | Comparative experiment | Mental health | Self-directed course for stress reduction and problem solving | Not mentioned | LINE messaging platform | Text, emojis, images | Not mentioned | Development platform: Heroku, Inc. server  Conversation developed using messaging API program provided by LINE | 27 college students  85 mins | CA was more effective than web-based course to decrease stress and improve self-esteem |
| Kowatsch 2017 (a) [72]  Kowatsch 2017 (b) [74]  L’Allemand 2018 [33]  Heldt 2018 [31]  Switzerland | RCT | Weight management | Lifestyle intervention for overweight adolescents | Anna & Lukas  Peer-like  Empathetic | Standalone app | Text, images | Daily challenges  Virtual rewards  Direct communication with parents & HCPs | Development platform: Mobile coach open-source software platform for the design and evaluation of mobile TBHCs  Development team: computer scientists, physicians, psychotherapist, diet and sport experts  Predefined text  Sensor integration to monitor physical activity | 22 overweight adolescents  12 months | Intermediate result reports of 13/15 participants:  High user compliance  67% participants interacted at least 4 times with the CA at 5.5 months |
| Kowatsch 2021 [73]  Switzerland | Multisite, single-arm, feasibility study | Asthma | Support asthma self-management by providing coaching sessions to increase patients’ cognitive and behavioral skills | Max  Empathetic  Supportive coach | Standalone app | Multimodal: Patients: Text-based CA (text, images, video  Parents: SMS  HCPs: email/ website  HCPs with parents and patients: face-to-face, phone call, text messages | App onboarding done by the HCP  Gamification (reward points that converted to lottery chances)  CA presented as HCP digital assistant to build working alliance  App usage reminders  Personalized coaching schedule | Development platform: Mobile coach open-source, secure software platform for the design and evaluation of mobile TBHCs  Development team: computer scientists, asthma experts, young patients, and their parents  Sources of information: literature on asthma management in children, information systems and technology acceptance research, working alliance with CAs, BCTs, and experiential learning theory. | 49 asthma patients 10-15 years old  60 days | CA was highly accepted  Improvement in cognitive and behavioral skills  Intervention completion rate: 75.5% |
| Ly 2017 [3]  Sweden | Pilot RCT | Mental Health | Positive psychology and CBT interventions to improve wellbeing levels and decrease subjective stress | Shim  Empathetic | Standalone app | Text | Daily check-ins  Tailored content  Summaries | Pre-written dialogs developed by psychologists  User responses: selecting options and free text  Database updated after user interactions to improve context in communication | 28 non-clinical adults  2 weeks | High participant engagement and adherence with intervention  Improvements in perceived stress and satisfaction with life in intervention group |
| Stasinaki 2018 [34]  Stasinaki 2021 [79]  Switzerland | RCT | Weight management | Self-management intervention for adolescent obesity with clinical support including diet, physical exercise, and stress management | PathMate2  Anna & Lukas | Standalone app | Text, images, videos  Direct verbal communication with HCP | Virtual rewards  Activity progress Daily encouragement  Communication with HCPs and coaches | Development platform: Mobile coach open-source software platform for the design and evaluation of mobile TBHCs  User responses: selecting options | 31 youth (10-18 years) with overweight or obesity  Intervention: 5.5 months  Maintenance: 1 year | No sustained decrease in BMI  Increased muscle mass, strength and agility and decreased body fat  Average daily app use 71.5% |
| Wang 2018 [75]  Hong Kong | Unsure  Two-group comparison | Smoking cessation | Social support to promote smoking cessation | Not mentioned | WeChat | Text, images, video | Announcements Individual and group reminders  Scheduled conversations  Moderate group conversations | Development platform: WeChat’s public Web API services.  Conversations are saved to provide context to new CA-user interactions  Able to conduct group and private conversations | 401 adult smokers  2 months | Participants in intervention had higher smoking cessation rates  Higher participation in conversations correlated with increased smoking cessation rates |

**BP**: Blood Pressure; **BCT**: Behavioral Change Technique; **BMI**: Body Mass Index; **CBT**: Cognitive Behavioral Therapy; **HCP**: Healthcare Provider; **RCT**: Randomized Controlled Trial; **SMS**: Short Message System; **TBHC**: Text-Based Health Care Chatbot
